# A systematic review and individual patient data meta-analysis to determine the effect of primaquine dose on efficacy, tolerability and safety in uncomplicated *Plasmodium vivax* malaria in Latin America

**DOI:** 10.1016/j.lana.2026.101634

**Published:** 2026-09-12

**Authors:** Kathy Nguyen, Anielle de Pina-Costa, Megha Rajasekhar, Nathália N. Chamma-Siqueira, André Daher, Marcelo U. Ferreira, Margarete do Socorro M. Gomes, Lilia Gonzalez-Ceron, Justin A. Green, Gavin C.K.W. Koh, Simone Ladeia-Andrade, Alejandro Llanos-Cuentas, Wuelton Marcelo Monteiro, Suaine C. Negreiros, Alexandre Macedo de Oliveira, Dhelio B. Pereira, Giselle M.R. Viana, José Luiz F. Vieira, Lina M. Zuluaga-Idarraga, Hyaa Hoseen M. Alatawi, Philippe J. Guerin, Julie A. Simpson, Marcus VG. Lacerda, Ric N. Price, André M. Siqueira, Robert J. Commons

**Affiliations:** a Global and Tropical Health Division, Menzies School of Health Research and Charles Darwin University, Darwin, Northern Territory, Australia; b Centre for Epidemiology and Biostatistics, Melbourne School of Population and Global Health, The University of Melbourne, Melbourne, Victoria, Australia; c Instituto Nacional de Infectologia Evandro Chagas, Fundação Oswaldo Cruz (Fiocruz), Rio de Janeiro, Brazil; d Centro de Pesquisa, Diagnóstico e Treinamento em Malária (CPD-Mal) da Fiocruz e da Secretaria de Vigilância em Saúde e Ambiente, Ministério da Saúde, Brazil; e Universidade Federal Fluminense, Niterói, Brazil; f Laboratório de Malária, Instituto Evandro Chagas, Ministério da Saúde do Brasil, Ananindeua, Pará State, Brazil; g Fiocruz Clinical Research Platform, Oswaldo Cruz Foundation (FIOCRUZ), Rio de Janeiro, Brazil; h Vice-presidency of Research and Biological Collections, Oswaldo Cruz Foundation (FIOCRUZ), Rio de Janeiro, Brazil; i Department of Parasitology, Institute of Biomedical Sciences, University of São Paulo, São Paulo, Brazil; j Global Health and Tropical Medicine, Institute of Hygiene and Tropical Medicine, NOVA University of Lisbon, Lisbon, Portugal; k Superintendência de Vigilância em Saúde do Estado do Amapá - SVS/AP, Macapá, Brazil; l Federal University of Amapá (Universidade Federal do Amapá - UNIFAP), Macapá, Brazil; m Regional Centre for Public Health Research, National Institute for Public Health, Tapachula, Chiapas, Mexico; n GARDP, Geneva, Switzerland; o Department of Infectious Diseases, Northwick Park Hospital, Harrow, UK; p Laboratory of Parasitic Diseases, Oswaldo Cruz Institute, Fiocruz, Rio de Janeiro, Brazil; q Unit of Leishmaniasis and Malaria, Instituto de Medicina Tropical “Alexander von Humboldt”, Universidad Peruana Cayetano Heredia, Peru; r Fundação de Medicina Tropical Dr Heitor Vieira Dourado, Manaus, Brazil; s Universidade do Estado do Amazonas, Manaus, Brazil; t Secretaria de Saúde do Estado do Acre, Cruzeiro do Sul, Brazil; u Malaria Branch, Division of Parasitic Diseases and Malaria, Center for Global Health, Centers for Disease Control and Prevention, Atlanta, USA; v Centro de Pesquisa em Medicina Tropical de Rondônia (CEPEM), Porto Velho, Brazil; w Fundação Universidade Federal de Rondonia (UNIR), Porto Velho, Brazil; x Centro Universitário do Estado do Pará - CESUPA, Belém, Brazil; y Programa de Pós-Graduação em Biologia de Agentes Infecciosos e Parasitários, Universidade Federal do Pará - UFPA, Belém, Brazil; z Federal University of Pará (Universidade Federal do Pará - UFPA), Belém, Brazil; aa Grupo Malaria, Facultad de Medicina, Universidad de Antioquia, Medellín, Colombia; ab Facultad Nacional de Salud Publica, Universidad de Antioquia, Medellin, Colombia; ac WorldWide Antimalarial Resistance Network (WWARN), Asia-Pacific Regional Centre, Melbourne, Australia; ad Centre for Tropical Medicine, Nuffield Department of Clinical Medicine, University of Oxford, Oxford, UK; ae Infectious Diseases Data Observatory (IDDO), Oxford, UK; af Instituto Leônidas & Maria Deane, Fiocruz, Manaus, Brazil; ag University of Texas Medical Branch, Galveston, USA; ah General and Subspecialty Medicine, Grampians Health - Ballarat, Ballarat, Australia

**Keywords:** *Plasmodium vivax*, Malaria, Primaquine, Recurrence, Latin America

## Abstract

**Background:**

Primaquine is widely used to prevent relapses of *Plasmodium vivax* malaria, but the optimal dose in Latin America is unknown. We undertook a systematic review and individual patient data meta-analysis to investigate the efficacy, tolerability and safety of different primaquine dosing regimens for the prevention of *P. vivax* recurrences in Latin America.

**Methods:**

MEDLINE, Web of Science, Embase, Scopus and Cochrane Central were systematically searched for prospective clinical efficacy studies of patients with uncomplicated *P. vivax* malaria in Latin America treated with daily primaquine within 28 days of schizontocidal treatment and published between January 1, 2000 and July 3, 2026. The efficacy of the total primaquine mg/kg dose on the rate of any *P. vivax* recurrence within 150 days of completing antimalarial treatment was assessed by Cox’s proportional hazards regression. The effect of the daily primaquine mg/kg dose on gastrointestinal symptoms was assessed 5–7 days after starting primaquine and on the risk of haemolysis on days 1–14 after starting primaquine. The systematic review was registered at PROSPERO CRD42024598742.

**Findings:**

Of 40 eligible studies, 1734 patients from 13 studies were included. The cumulative incidence of recurrence 150 days after the last dose of primaquine was 68.2% (95% CI 59.5–76.6) in 119 patients not treated with primaquine, 30.8% (27.0–35.0) in 1000 patients treated with low total dose (2 to <5 mg/kg) primaquine, and 8.7% (5.6–13.2) in 286 patients treated with high total dose (≥5 mg/kg) primaquine. The rate of recurrence within 150 days was lower in patients treated with high compared to low total dose primaquine (adjusted hazard ratio (AHR) 0.36, 95% CI 0.21–0.62; p < 0.0001). Gastrointestinal disturbance was reported in 1.1% (1/94) of patients administered a low daily primaquine dose (<0.375 mg/kg/day) and 2.6% (4/152) administered an intermediate daily dose (0.375 to <0.75 mg/kg/day). None of 501 patients with ≥30% G6PD activity had an acute haemoglobin drop by >25% to <7 g/dL within 14 days of starting primaquine, although only 7 patients received high daily dose primaquine (≥0.75 mg/kg/day).

**Interpretation:**

Patients treated with high total dose primaquine had less than half the risk of *P. vivax* recurrence compared to those treated with low dose primaquine. The high dose regimen was well tolerated and no safety concerns were observed in patients with ≥30% G6PD activity.

**Funding:**

Bill and Melinda Gates Foundation, Australian National Health and Medical Research Council and Royal Australasian College of Physicians.

**Research in context**

**Evidence before this study**

World Health Organization guidelines recommend the use of high total dose primaquine (7 mg/kg) in most *Plasmodium* *vivax*-endemic malaria regions globally, however, a low total dose (3.5 mg/kg) regimen is still recommended for Latin America. We searched MEDLINE, Embase, Scopus, Web of Science, and the Cochrane Database of Systematic reviews for randomised controlled trials of uncomplicated *P. vivax* malaria in Latin America comparing high and low total dose primaquine published between January 1, 2000 and January 30, 2026 in any language using the search terms ‘vivax’, ‘primaquine’, and ‘America’. Two trials have suggested that the risk of recurrence was lower following high dose primaquine compared with low dose primaquine, but one delayed the administration of primaquine and the other was stopped early.

**Added value of this study**

This systematic review and individual patient data meta-analysis of data from 1734 patients from 13 studies found that the rate of *P. vivax* recurrence within 150 days of completing primaquine was more than halved following high total dose primaquine compared with low total dose primaquine. High and low dose primaquine regimens were well tolerated with no safety concerns observed in patients with 30% G6PD activity or more.

**Implications of all the available evidence**

Consistent with other *P. vivax*-endemic regions, high total dose primaquine regimens are associated with substantial reductions in the risk of recurrence compared with low dose regimens in Latin America, supporting their use in patients who have undergone G6PD testing.

## Introduction

Between 2000 and 2015 the annual number of *P. vivax* malaria cases in Latin America declined from ∼1.1 million to 402,000. However, the reduction has since plateaued, with 419,000 cases in 2024.^1^ The majority of malaria cases on the continent occur in three countries (Venezuela, Brazil, and Colombia) situated within the Amazon Basin, where ecological and socio-environmental conditions favour malaria transmission.^1^

*P. vivax* relapses are caused by activation of hypnozoites in the liver, weeks or months after the initial infection.^2^ Relapses can cause symptomatic illness and a cumulative risk of anaemia and associated morbidity.^3^^,^^4^ Effective *P. vivax* treatment requires radical cure of all stages of the parasite, including treatment of the blood stages with a schizontocidal agent, such as chloroquine or an artemisinin-based combination therapy (ACT), plus treatment of the liver stages with a hypnozoitocide, such as primaquine or tafenoquine.^5^ Global World Health Organization (WHO) antimalarial guidelines currently recommend a total primaquine dose of 3.5 mg/kg for Latin America, administered once daily over 7 or 14 days.^6^ In Brazil, patients with recurrent *P. vivax* malaria within 60 days of initial treatment are retreated with a higher total dose of primaquine (7 mg/kg) over 14 days.^7^ Single-dose tafenoquine has begun to be implemented in some national policies.^8^

Patients with glucose-6-phosphate dehydrogenase (G6PD) deficiency are at risk of serious haemolysis when treated with primaquine or tafenoquine. Antirelapse efficacy of primaquine is determined by the total dose administered, whereas tolerability and the risk of haemolysis are associated with the daily dose.^9^^,^^10^ Regimens of high total dose (7 mg/kg) primaquine administered over 7 or 14-days have been recommended for patients with ≥70% and ≥30% G6PD activity, respectively, and tafenoquine is recommended for patients with ≥70% G6PD activity.^6^ Hence, the use of higher daily dose primaquine and tafenoquine requires testing of G6PD activity prior to treatment to reduce the risk of haemolysis.^11^

Recent global studies found that patients treated with a high total primaquine dose have 55% lower risk of recurrence compared to patients treated with a low total dose (3.5 mg/kg).^9^^,^^10^ However, transmission intensity, hypnozoite load and the prevalence of G6PD deficiency differ significantly between different *P. vivax*-endemic settings and thus a higher dose may not be necessary in all endemic areas.^6^^,^^12^^,^^13^ Furthermore, despite implementation of tafenoquine for patients with normal (≥70%) G6PD activity in Brazil,^8^ almost a third of patients have lower G6PD activity and thus require primaquine to achieve radical cure. We undertook an individual patient data (IPD) meta-analysis of primaquine efficacy studies from Latin America to determine the effect of primaquine dose on efficacy, tolerability, and safety, and to inform local antimalarial treatment guidelines.

## Methods

### Search strategy and selection criteria

We searched MEDLINE, Web of Science, Embase, Scopus and Cochrane Central for clinical efficacy studies of patients with uncomplicated *P. vivax* monoinfections published in any language between January 1, 2000 and July 3, 2026 (Appendix Text S1). The systematic review was registered at PROSPERO CRD42024598742 and is presented according to the PRISMA IPD statement (Appendix Checklist S1).

Studies were included if they were prospective clinical efficacy studies conducted in Latin America (all countries south of and including Mexico on the American continent) with a minimum active follow-up of 28 days following presentation. Patients had to be treated with chloroquine or one of six common ACTs, with at least one treatment arm with daily primaquine administered over multiple days and commencing within 28 days of schizontocidal treatment (Appendix Text S1).

### Data collation

Investigators of eligible studies were invited to share IPD, and to contribute additional data from eligible unpublished studies where data were available by July 26, 2024. IPD were not requested for studies published between July 26, 2024 and July 3, 2026 due to the long time required to obtain approvals and collate the data. Shared data were pseudonymised and collated using standardised methodology in the WorldWide Antimalarial Resistance Network (WWARN) repository, part of the Infectious Diseases Data Observatory (IDDO).^14^

Patients were excluded if data were missing for age, sex, weight, baseline parasitaemia, or total mg/kg dose of primaquine. Patients were excluded if, on presentation, they were pregnant, lactating, or had severe malaria, if they were treated with intermittent primaquine regimens, or if they received unplanned adjunctive antimalarial treatments after initial treatment. Efficacy, tolerability and safety analyses were further restricted (Appendix Text S1).

### Outcomes

The primary efficacy outcome was any *P. vivax* recurrence (symptomatic or asymptomatic) occurring between the last dose of primaquine and 150 days after the last dose of primaquine as described in Appendix Text S1. Secondary efficacy outcomes included: any *P. vivax* recurrence between the last primaquine dose and 90 days after the last primaquine dose and any symptomatic *P. vivax* recurrence between the last primaquine dose and 150 days after the last primaquine dose.

The primary tolerability outcome was the incidence of any gastrointestinal disturbance (a composite endpoint of any of vomiting, anorexia or diarrhoea) on days 5–7 after starting primaquine. Secondary tolerability outcomes were the incidence of the composite gastrointestinal endpoint on day 1 and/or day 2 after starting primaquine and the incidence of any vomiting 1 h after primaquine dosing (i.e., acute vomiting) at two separate timepoints: days 0–2 and days 3–14 after starting primaquine.^9^

The primary safety outcome was a reduction in haemoglobin concentration greater than 25% to a concentration less than 7 g/dL between starting primaquine and days 1–14 after starting primaquine.^15^ Secondary safety outcomes were: (1) the maximum absolute reduction of haemoglobin on day 2 and/or day 3 after starting primaquine (the expected nadir^15^); (2) a composite outcome of the following: haemoglobin fall below 5 g/dL or haemoglobin fall >5 g/dL from 1 to 14 days after starting primaquine, renal failure needing dialysis, blood transfusion or death within 1–28 days of starting primaquine; and (3) the development of new-onset mild, moderate or severe anaemia within 2–3 days of starting primaquine. Safety outcomes were assessed in patients with G6PD activity of ≥30%.

### Data analysis

The primaquine mg/kg dose exposure was calculated from the number of tablets administered to each patient. If the number of daily tablets was unavailable, dosing was derived from the patient’s weight and the dosing schedule in each study protocol. For the efficacy analysis, total primaquine dose was categorised into very low (<2 mg/kg), low (2 to <5 mg/kg, reflecting a target dose of 3.5 mg/kg) and high dose (≥5 mg/kg, reflecting a target dose of 7 mg/kg).^9^ For tolerability and safety analyses, daily primaquine dose was categorised as low dose (<0.375 mg/kg/day), intermediate (≥0.375 to <0.75 mg/kg/day) and high dose (≥0.75 mg/kg/day).^10^ Primaquine duration was categorised as 7 days or 14 days.

Day 0 was defined as the day that treatment with a schizontocidal antimalarial was commenced. For efficacy analyses, days after the last primaquine dose were defined as the number of days after the expected completion of the primaquine regimen. For tolerability and safety analyses, days after starting primaquine were defined as the number of days after the first day of the primaquine regimen. If primaquine was not administered, the equivalent day was calculated relative to the first or last day of primaquine treatment in the concurrent primaquine arm of that respective study (Appendix Fig. S1).

The cumulative incidence of *P. vivax* recurrence between the last primaquine dose and the efficacy endpoints were calculated using Kaplan–Meier survival analysis, stratified by primaquine treatment groups. Cox's proportional hazards regression was used to estimate the effect of the total primaquine mg/kg dose on the time to first *P. vivax* recurrence between the last primaquine dose and the efficacy endpoints. Based on a causal diagram (directed acyclic graph), the model was adjusted for age, sex and baseline parasitaemia, with shared frailty for study site (Appendix Fig. S2). The proportional hazards assumption was checked visually using log–log survival plots.

The number and proportion of patients with categorical primary and secondary tolerability and safety outcomes were calculated according to the daily primaquine dose groups. The denominator for tolerability analyses was defined as the number of patients who had at least one of the symptoms assessed at that time point. The mean (standard deviation) change in haemoglobin concentration between the initial primaquine treatment and the minimum measurement on days 2–3 after starting primaquine was calculated according to daily primaquine dose groups.

Studies were assessed for risk of bias using the Cochrane Risk of Bias 2 tool^16^ and the Joanna Briggs Institute Case Series tool.^17^ The baseline characteristics of included studies were compared with studies that were eligible but not included to assess for inclusion bias. Further details of analyses, including sensitivity analyses are described in the Appendix Text S1.

### Ethics approval statement

Shared data were obtained according to ethical approvals from the country of origin and the original study. The data were deidentified and cannot be linked to individuals. As such, our analysis did not require additional ethics approval according to the guidelines of Oxford Central University Ethics Committee. This activity was reviewed by CDC, deemed not research, and was conducted consistent with applicable federal law and CDC policy.

### Role of funding source

The funders of the study had no role in study design, data collection, analysis, interpretation, or writing of the report.

## Results

**Fig. 1:**
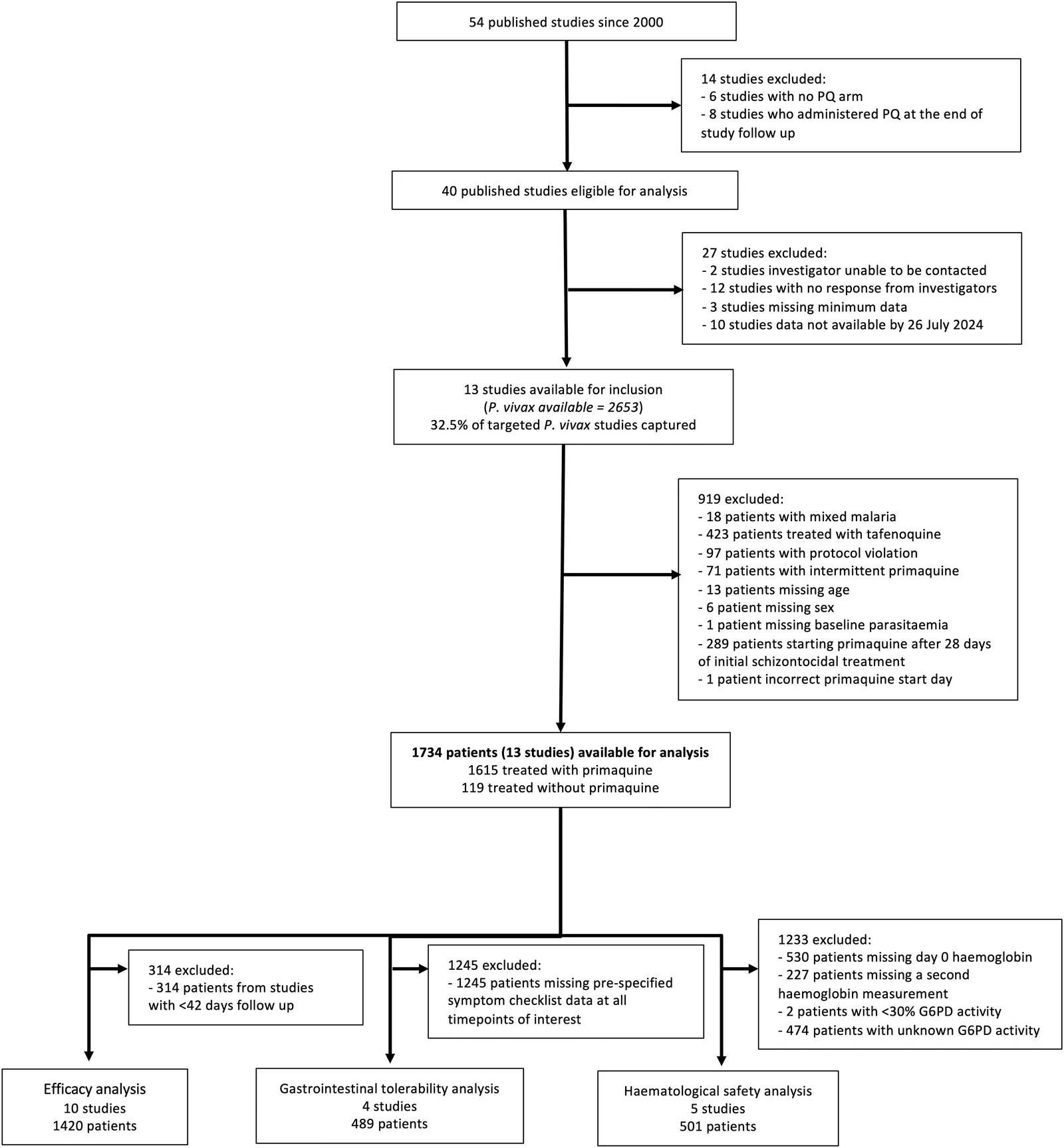
Study selection of *P. vivax* clinical efficacy studies from Latin America from 2000 to 2024.

Between January 1, 2000 and July 3, 2026, 54 *P. vivax* efficacy studies enrolling patients in Latin America were published, of which 40 were eligible for inclusion (Fig. 1). Of these 40 studies, 27 were excluded (Appendix Tables S1 and S2). Individual patient data were available from 13 published studies (2653 patients).^18^, ^19^, ^20^, ^21^, ^22^, ^23^, ^24^, ^25^, ^26^, ^27^, ^28^, ^29^, ^30^ After exclusion of 919 patients, 1734 patients from 13 studies (8 randomised controlled studies and 5 observational studies) were included in the analysis (Fig. 1, Appendix Tables S1–S6).^18^, ^19^, ^20^, ^21^, ^22^, ^23^, ^24^, ^25^, ^26^, ^27^, ^28^, ^29^ Twelve studies had low or unclear risk of bias (Appendix Tables S7 and S8).

A total of 1420 patients from 10 studies were included in the efficacy analysis. The median patient age was 32.1 years (interquartile range [IQR] 19.0–45.0), with a median weight of 64.0 kg (IQR 54.0–75.0; Table 1), 64.7% of patients were male and 79.7% were treated with chloroquine (Table 1). Overall, 1301 (91.6%) patients were treated with primaquine and 119 (8.4%) were treated without primaquine. Of the patients treated with primaquine, 15 (1.1%) received very low total dose primaquine, 1000 (70.4%) received low dose primaquine and 286 (20.4%) received high dose primaquine. Of the 1301 patients treated with daily primaquine, 868 (66.7%) took the first dose of primaquine on day 0. Patients treated with high dose primaquine more frequently came from regions of higher transmission intensity, had higher *P. vivax* baseline parasite density, were administered dihydroartemisinin-piperaquine as a schizontocidal agent, had their primaquine dose unsupervised and had total primaquine dose derived from actual dosing rather than planned dose, compared to other treatment groups (Table 1). Of the 15 patients treated with very low total dose primaquine, 12 (80%) ceased treatment and were lost to follow up early; this treatment category was therefore excluded from subsequent analyses.

**Table 1:** Demographic and baseline characteristics for patients included in the efficacy analysis of *P. vivax* clinical efficacy studies from Latin America from 2000 to 2024.

	Total	No primaquine	Very low dose total primaquine (<2 mg/kg)	Low dose total primaquine (2 to <5 mg/kg)	High dose total primaquine (≥5 mg/kg)
	N = 1420	N = 119	N = 15	N = 1000	N = 286
Age (years)					
Median	32.1 (19.0–45.0)	35.0 (26.0–49.0)	30.0 (25.0–42.0)	32.0 (18.0–44.0)	33.4 (19.0–49.0)
<5	38/1420 (2.7%)	0/119 (0.0%)	0/15 (0.0%)	31/1000 (3.1%)	7/286 (2.4%)
5 to <15	210/1420 (14.8%)	0/119 (0.0%)	2/15 (13.3%)	168/1000 (16.8%)	40/286 (14.0%)
≥15	1172/1420 (82.5%)	119/119 (100.0%)	13/15 (86.7%)	801/1000 (80.1%)	239/286 (83.6%)
Sex					
Male	919/1420 (64.7%)	82/119 (68.9%)	12/15 (80.0%)	658/1000 (65.8%)	167/286 (58.4%)
Female	501/1420 (35.3%)	37/119 (31.1%)	3/15 (20.0%)	342/1000 (34.2%)	119/286 (41.6%)
Enrolment variables					
Weight (kg)	64.0 (54.0–75.0)	64.5 (60.2–72.5)	80.5 (67.0–90.0)	63.3 (53.0–75.0)	65.3 (54.0–76.0)
Presence of recent fever	1080/1156 (93.4%)	111/119 (93.3%)	12/15 (80.0%)	672/736 (91.3%)	285/286 (99.7%)
Parasitaemia (parasites/mL)	3171.5 (1107.7–6067.5)	4750.0 (1633.0–10243.0)	2698.3 (576.0–4264.0)	2991.9 (1054.2–5590.0)	3975.2 (1264.0–6612.3)
Haemoglobin (g/dL)^a^	13.2 (1.7)	12.9 (1.4)	14.3 (1.7)	13.1 (1.8)	13.6 (1.7)
Schizontocidal treatment					
Artemether-lumefantrine	87/1420 (6.1%)	0/119 (0.0%)	0/15 (0.0%)	87/1000 (8.7%)	0/286 (0.0%)
Artesunate-mefloquine	89/1420 (6.3%)	0/119 (0.0%)	0/15 (0.0%)	89/1000 (8.9%)	0/286 (0.0%)
Chloroquine	1132/1420 (79.7%)	119/119 (100.0%)	10/15 (66.7%)	803/1000 (80.3%)	200/286 (69.9%)
Dihydroartemisinin-piperaquine	112/1420 (7.9%)	0/119 (0.0%)	5/15 (33.3%)	21/1000 (2.1%)	86/286 (30.1%)
Primaquine dosing					
Total dose (mg/kg)	3.5 (3.1–4.6)		1.1 (0.7–1.4)	3.3 (3.1–3.7)	6.5 (5.6–7.1)
Primaquine start day					
Day 0–2	1049/1301 (80.6%)		15/15 (100.0%)	847/1000 (84.7%)	187/286 (65.4%)
Day 3–14	63/1301 (4.8%)		0/15 (0.0%)	39/1000 (3.9%)	24/286 (8.4%)
>Day 14	189/1301 (14.5%)		0/15 (0.0%)	114/1000 (11.4%)	75/286 (26.2%)
Primaquine duration					
7 days	627/1300 (48.2%)		2/15 (13.3%)	614/999 (61.5%)	11/286 (3.8%)
14 days	673/1300 (51.8%)		13/15 (86.7%)	385/999 (38.5%)	275/286 (96.2%)
Total dose of primaquine calculation method					
Actual dosing	851/1301 (65.4%)		14/15 (93.3%)	554/1000 (55.4%)	283/286 (99.0%)
Protocol dosing	450/1301 (34.6%)		1/15 (6.7%)	446/1000 (44.6%)	3/286 (1.0%)
Primaquine dosing supervision					
Unsupervised	285/1301 (21.9%)		12/15 (80.0%)	102/1000 (10.2%)	171/286 (59.8%)
Partially supervised	558/1301 (42.9%)		3/15 (20.0%)	550/1000 (55.0%)	5/286 (1.7%)
Fully supervised	458/1301 (35.2%)		0/15 (0.0%)	348/1000 (34.8%)	110/286 (38.5%)
Study duration (days)					
42	113/1420 (8.0%)	0/119 (0.0%)	2/15 (13.3%)	106/1000 (10.6%)	5/286 (1.7%)
63	264/1420 (18.6%)	0/119 (0.0%)	0/15 (0.0%)	264/1000 (26.4%)	0/286 (0.0%)
168	250/1420 (17.6%)	0/119 (0.0%)	0/15 (0.0%)	152/1000 (15.2%)	98/286 (34.3%)
180	705/1420 (49.6%)	119/119 (100.0%)	13/15 (86.7%)	400/1000 (40.0%)	173/286 (60.5%)
365	88/1420 (6.2%)	0/119 (0.0%)	0/15 (0.0%)	78/1000 (7.8%)	10/286 (3.5%)
Primaquine administered with food					
No	344/1077 (31.9%)		0/3 (0.0%)	245/957 (25.6%)	99/117 (84.6%)
Yes	294/1077 (27.3%)		3/3 (100.0%)	286/957 (29.9%)	5/117 (4.3%)
Recommended	439/1077 (40.8%)		0/3 (0.0%)	426/957 (44.5%)	13/117 (11.1%)
Transmission intensity^b^					
Low	88/1420 (6.2%)	0/119 (0.0%)	0/15 (0.0%)	78/1000 (7.8%)	10/286 (3.5%)
Moderate	206/1420 (14.5%)	0/119 (0.0%)	2/15 (13.3%)	196/1000 (19.6%)	8/286 (2.8%)
High	1126/1420 (79.3%)	119/119 (100.0%)	13/15 (86.7%)	726/1000 (72.6%)	268/286 (93.7%)
G6PD status^c^					
Unknown	776/1420 (54.6%)	0/119 (0.0%)	14/15 (93.3%)	575/1000 (57.5%)	187/286 (65.4%)
<30%	2/1420 (0.1%)	0/119 (0.0%)	0/15 (0.0%)	2/1000 (0.0%)	0/286 (0.0%)
≥30%	642/1420 (45.4%)	119/119 (100.0%)	1/15 (6.7%)	423/1000 (42.3%)	99/286 (34.6%)
Countries					
Brazil	1085/1420 (76.4%)	58/119 (48.7%)	15/15 (100.0%)	739/1000 (73.9%)	273/286 (95.5%)
Colombia	93/1420 (6.5%)	0/119 (0.0%)	0/15 (0.0%)	90/1000 (9.0%)	3/286 (1.0%)
Mexico	88/1420 (6.2%)	0/119 (0.0%)	0/15 (0.0%)	78/1000 (7.8%)	10/286 (3.5%)
Peru	154/1420 (10.8%)	61/119 (51.3%)	0/15 (0.0%)	93/1000 (9.3%)	0/286 (0.0%)

Data are n (%), median (IQR), mean (SD) or n/N (%). G6PD = glucose-6-phosphate dehydrogenase.

a If haemoglobin was not measured, haematocrit was converted to haemoglobin using the formula: haemoglobin (g/dL) = (haemtocrit [%] – 5.62)/2.60.

b Transmission intensity of study sites was classified as low (<1 case per 1000 person-years), moderate (1 case to <10 cases per 1000 person-years), and high (≥10 cases per 1000 person-years) on the basis of subnational malaria incidence estimates for the median year of enrolment.

c G6PD deficiency was categorised as deficient (<30% activity or an abnormal qualitative test) and normal (≥30% activity or a normal qualitative test).

**Fig. 2:**
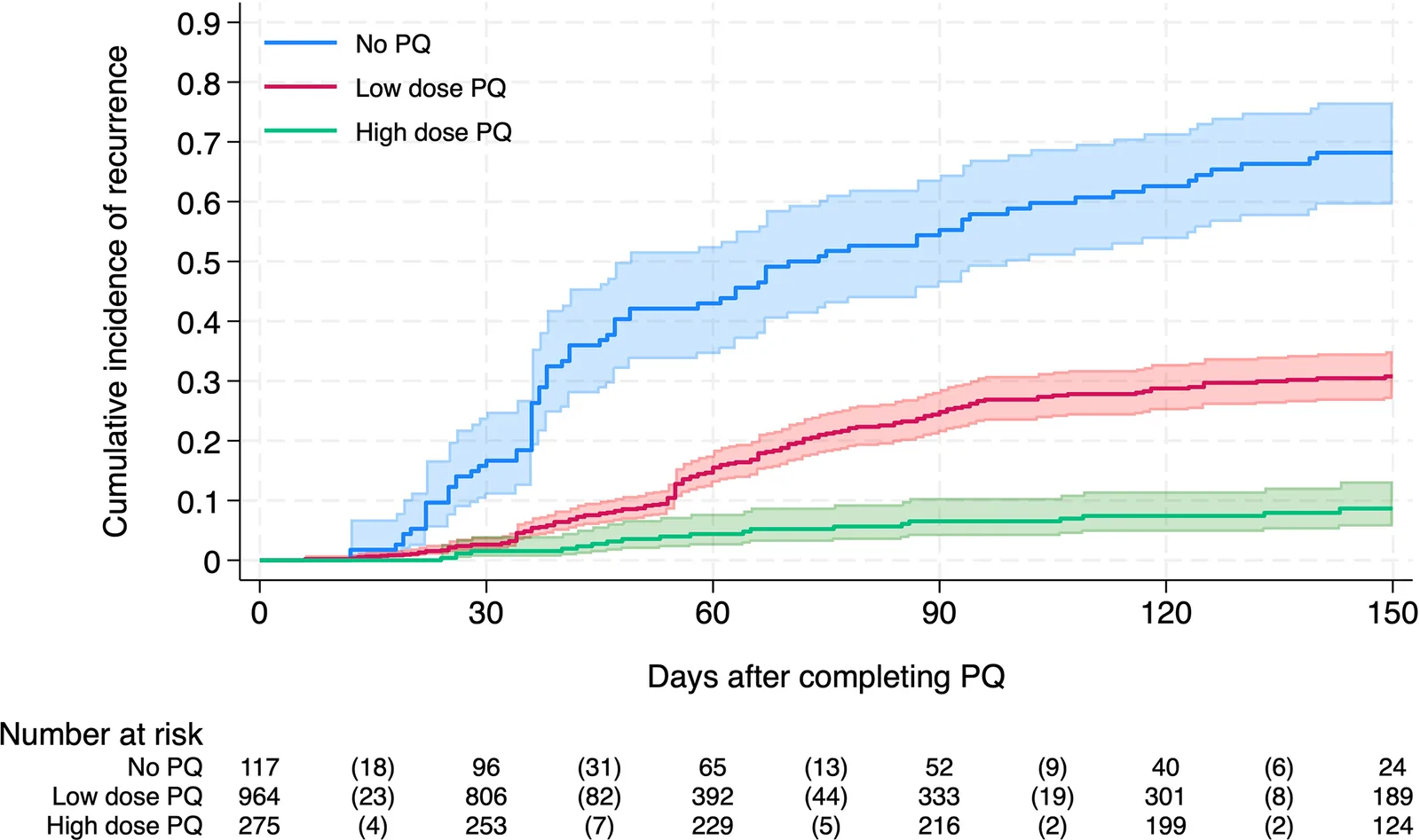
Cumulative incidence of first *Plasmodium vivax* parasitaemia in patients between the day after the last expected primaquine dose and 150 days later by primaquine total dose in *P. vivax* clinical efficacy studies from Latin America from 2000 to 2024. PQ, primaquine; Primaquine categorised as low total mg/kg dose (<5 mg/kg) and high total mg/kg dose (≥5 mg/kg).

At day 150 after the last primaquine dose, the cumulative incidence of *P. vivax* recurrence was 68.2% (95% confidence interval (CI) 59.5–76.6) in patients not treated with primaquine, 30.8% (27.0–35.0) following low total dose primaquine and 8.7% (5.6–13.2) following high total dose primaquine (Fig. 2, Appendix Fig. S3). At day 90 after the last primaquine dose, the corresponding cumulative incidences of *P. vivax* recurrence were 55.3% (95% CI 46.4–64.5), 24.8% (21.4–28.7) and 6.5% (4.0–10.4), respectively.

After adjusting for confounders, the rate of recurrence for any parasitaemia within 150 days of the last primaquine dose was lower in patients treated with high total dose primaquine compared with low total dose primaquine (adjusted hazard ratio (AHR) 0.36, 95% CI 0.21–0.62). Compared to patients not treated with primaquine, the AHR was 0.33 (0.23–0.49) following low total dose primaquine and 0.12 (0.06–0.23) following high total dose primaquine. Results for symptomatic recurrences were similar; the rate of symptomatic recurrences over 150 days after the last dose of primaquine was lower following high total dose compared to low total dose primaquine (AHR 0.23, 0.09–0.60).

The magnitude of effect observed with high total dose primaquine remained consistent in the sensitivity analyses, which included variations in schizontocidal partner drugs, study inclusion and timing of primaquine initiation (Appendix Tables S9 and S10, Appendix Figs. S4 and S5). The events and person-time for each study are shown in the Appendix Table S11. Only one included study directly compared high versus low total dose primaquine,^29^ while two studies compared low total dose primaquine with no primaquine (Appendix Fig. S6)^18^^,^^26^ and no studies compared high total dose primaquine with no primaquine.

Of 1000 patients treated with a low total dose of primaquine, 20.2% (78/386) treated with a 14-day regimen had recurrent *P. vivax* parasitaemia by day 150 after the last primaquine dose compared to 16.0% (98/614) of those treated with a 7-day regimen. Of 614 patients treated with 7-day low dose primaquine, 71.3% (438) were co-treated with chloroquine, compared with 94.6% (365/386) of patients treated with 14-day low dose primaquine. In 803 patients treated with chloroquine and low total dose primaquine, the adjusted rate of recurrence was lower in those receiving a 14-day regimen compared to a 7-day regimen (AHR 0.39, 95% CI 0.16–0.94; Appendix Fig. S6). However, only one study^29^ contributed to the cumulative incidence after day 55 after the last primaquine dose in patients with a 7-day regimen, and sensitivity analyses found estimates were impacted by exclusion of individual studies, reducing the generalisability of these results (Appendix Table S12 and Figs. S7 and S8). Although this analysis was pre-planned, the available data do not allow a thorough examination of this question; hence we consider this comparison exploratory. Similar analyses comparing 7- and 14-day regimens could not be conducted in patients treated with high total dose primaquine since 96.1% (275/286) were treated with a 14-day regimen.

Overall 489 patients from four studies were eligible for inclusion in the tolerability analysis (Appendix Table S13); 173 (35.4%) were treated with a low daily dose of primaquine (<0.375 mg/kg), 309 (63.2%) with an intermediate daily dose of primaquine (≥0.375 and <0.75 mg/kg) and 7 (1.4%) with a high daily dose of primaquine (≥0.75 mg/kg; Table 2). All patients started primaquine treatment on day 0.

**Table 2:** Gastrointestinal tolerability outcomes in *P. vivax* clinical efficacy studies from Latin America from 2000 to 2024.

	Total	Low daily dose primaquine (<0.375 mg/kg/day)	Intermediate daily dose primaquine (≥0.375 & <0.75 mg/kg/day)	High daily dose primaquine (≥0.75 mg/kg/day)
	N = 489	N = 173	N = 309	N = 7
Days 5–7				
Composite	5/250 (2.0%)	1/94 (1.1%)	4/152 (2.6%)	0/4 (0.0%)
Vomiting	2/161 (1.2%)	0/92 (0.0%)	2/69 (2.9%)	–
Anorexia	1/173 (0.6%)	0/77 (0.0%)	1/92 (1.1%)	0/4 (0.0%)
Diarrhoea	3/250 (1.2%)	1/94 (1.1%)	2/152 (1.3%)	0/4 (0.0%)
Days 1–2				
Composite	124/274 (45.3%)	38/103 (36.9%)	84/167 (50.3%)	2/4 (50.0%)
Vomiting	30/183 (16.4%)	15/101 (14.9%)	15/82 (18.3%)	–
Anorexia	92/189 (48.7%)	27/85 (31.8%)	63/100 (63.0%)	2/4 (50.0%)
Diarrhoea	38/273 (13.9%)	16/103 (15.5%)	22/166 (13.3%)	0/4 (0.0%)
Day 0				
Composite	361/489 (73.8%)	136/173 (78.6%)	221/309 (71.5%)	4/7 (57.1%)
Vomiting	137/395 (34.7%)	71/171 (41.5%)	65/221 (29.4%)	1/3 (33.3%)
Anorexia	321/488 (65.8%)	114/173 (65.9%)	203/308 (65.9%)	4/7 (57.1%)
Diarrhoea	45/489 (9.2%)	14/173 (8.1%)	30/309 (9.7%)	1/7 (14.3%)

Composite outcome is defined as experiencing any vomiting, diarrhoea or anorexia on day of outcome.

Gastrointestinal symptoms 5–7 days after starting primaquine were reported in 2.0% (5/250) of patients: 1.0% (1/94) in those treated with a low daily dose, 2.6% (4/152) following an intermediate daily dose and none (0/4) following a high daily dose. Reporting of gastrointestinal disturbance was greatest during early primaquine treatment: 73.8% (381/489) on the day starting primaquine and 45.3% (124/274) 1–2 days after starting primaquine (Table 2). There were no reports of acute vomiting (0/489 patients) within 1 h of primaquine administration on days 0–2 following any dose of primaquine.

In total, 501 patients from five studies with ≥30% G6PD activity were eligible for inclusion in the safety analysis, of whom 119 (23.8%) were not treated with primaquine, 129 (25.7%) were treated with low daily dose primaquine, 246 (49.1%) with intermediate daily dose primaquine, and 7 (1.4%) with high daily dose primaquine (Appendix Table S14).

None of the patients had an acute drop in haemoglobin concentration of >25% to <7 g/dL within 14 days of starting primaquine. Of 244 patients with haemoglobin measured on days 1–14, one (0.4%) treated with an intermediate daily dose of primaquine (0.47 mg/kg/day) had a >5 g/dL drop in haemoglobin concentration from 17.5 g/dL at baseline to 12.2 g/dL on day 2 after starting primaquine (Table 3). No patients required a blood transfusion, had a fall in haemoglobin to less than 5 g/dL or developed renal failure requiring dialysis.

**Table 3:** Haematological safety outcomes in patients with glucose-6-phosphate-dehydrogenase activity ≥30% in *P. vivax* clinical efficacy studies from Latin America from 2000 to 2024.

Haemoglobin outcome	Total	No primaquine	Low daily dose primaquine (<0.375 mg/kg/day)	Intermediate daily dose primaquine (≥0.375 & <0.75 mg/kg/day)	High daily dose primaquine (≥0.75 mg/kg/day)
	N = 501	N = 119	N = 129	N = 246	N = 7
>25% reduction from baseline to <7 g/dL by day 14^a^	0/499 (0.0%)	0/119 (0.0%)	0/129 (0.0%)	0/244 (0.0%)	0/7 (0.0%)
>5 g/dL reduction from baseline to day 14^a^	1/499 (0.2%)	0/119 (0.0%)	0/129 (0.0%)	1/244 (0.4%)	0/7 (0.0%)
Anaemia developed by days 2 or 3 after starting primaquine					
No	206/228 (90.4%)	91/104 (87.5%)	50/50 (100.0%)	62/70 (88.6%)	3/4 (75.0%)
Mild (≥8 to <11 g/dL)	22/228 (9.6%)	13/104 (12.5%)	0/50 (0.0%)	8/70 (11.4%)	1/4 (25.0%)
Reduction between day 0 and days 2–3 after starting primaquine					
Relative reduction in Hb (%)	1.7 (7.6)	2.8 (6.5)	−1.3 (7.8)	2.5 (8.3)	0.9 (6.6)
Absolute reduction in Hb (g/dL)	0.3 (1.0)	0.4 (0.8)	−0.1 (1.0)	0.4 (1.1)	0.1 (0.8)

n/N (%), mean (sd).

a Days after starting primaquine.

Mild anaemia (Hb ≥8 g/dL and <11 g/dl) that developed by days 2–3 occurred in 12.5% (13/104) of patients not treated with primaquine, 0% (0/50) treated with low daily dose primaquine, 11.4% (8/70) with intermediate daily dose primaquine and 25.0% (1/4) with high daily dose primaquine. The mean absolute and relative reductions in haemoglobin within 2–3 days of starting primaquine were small and similar across dosing groups (Table 3).

One male and one female patient had G6PD activity <30% and received a low daily dose of primaquine for 14 days. The male patient had a reduction in haemoglobin from 15.3 g/dL on day 0 to 12.3 g/dL on day 7 and the female patient had a reduction from 10.1 g/dL on day 0 to 7.1 g/dL on day 7. Neither patient ceased primaquine.

## Discussion

Primaquine remains the most widely used drug in Latin America to kill hypnozoites and prevent relapsing *P. vivax* malaria.^1^ Our IPD meta-analysis included data from 13 efficacy studies, and is the largest study to date to assess the benefits and risks of different primaquine regimens in Latin America. The results demonstrate a large difference in the risk of recurrent malaria between patients treated with high total dose primaquine (7 mg/kg) and those treated with low total dose (3.5 mg/kg), consistent with findings from a global analysis on primaquine efficacy.^9^ Importantly, higher daily doses of primaquine were not associated with gastrointestinal intolerance and there were no serious haemolytic events observed in individuals with ≥30% G6PD activity. Our findings have direct implications for malaria control programmes in Latin America, addressing the uncertainty highlighted in recent WHO guidelines,^6^ regarding the applicability of high total dose primaquine in the region.

In 2024, the WHO changed its global antimalarial treatment guidelines to recommend a high total primaquine dose in most *P. vivax* endemic countries.^6^^,^^9^ However, the certainty of evidence in the Americas was very low, and the revised WHO guidelines continued to recommend low dose primaquine for the region.^6^ Our current analysis support that the benefits of high dose primaquine extend to Latin America as well, with an estimated reduction in the risk of recurrences of 64% (AHR 0.36, 95% CI 0.21–0.62). Although there have been limited randomised comparisons of high versus low total dose primaquine in the Americas, our results are consistent with two trials comparing low and high total dose primaquine, one of which was included in our pooled analysis^29^ and one, which was stopped early due to greater efficacy with high total dose primaquine, that was not available at the time our data were collated (Appendix Fig. S9).^31^

Achieving a higher total primaquine dose would require extending the current regimen of 0.5 mg/kg/day from 7 to 14 days or increasing the daily dose to 1 mg/kg/day for 7 days. Higher primaquine daily doses of 0.5–1 mg/kg/day are associated with a greater risk of haemolysis in G6PD-deficient patients than 0.25 mg/kg/day, an important caveat in settings where screening for G6PD deficiency is not readily available. However, point of care quantitative G6PD screening has recently become available and is rolled out in some areas to support the use of tafenoquine. Like tafenoquine, the 1 mg/kg/day dose over 7 days is reserved for patients with activity ≥70% due to concerns of haemolysis, but primaquine at a daily dose of 0.5 mg/kg/day administered over 14 days can be used in patients with G6PD activity ≥30%.^6^ In settings where G6PD deficiency is very rare and resources are limited, policymakers may consider primaquine regimens of 0.5 mg/kg/day without G6PD testing, however, this increases the risk of serious haemolysis and careful patient education and monitoring would need to be integrated into malaria management.

The efficacy of 7- versus 14-day primaquine treatment regimens was compared in patients treated with a similar total dose. In patients treated with a low total dose of primaquine (3.5 mg/kg) in combination with chloroquine, there was a reduced risk of recurrence within 150 days of treatment with a 14-day regimen compared with a 7-day regimen. These results contrast with two previous large randomised controlled trials in which the 7-day 1 mg/kg/day dose regimen was non inferior to the 14-day 0.5 mg/kg/day regimen patients treated with high dose primaquine.^32^^,^^33^ In our pooled analysis the difference was observed in patients treated with a low dose regimen (3.5 mg/kg) but the results relied on a single study^29^ that provided data on the 7-day regimen after day 55 and results of the sensitivity analyses were inconsistent. Another study^27^ assessing the 7-day regimen only followed patients passively after day 28, potentially introducing bias into the reporting of recurrent parasitaemia. A similar comparison between 7- and 14-day regimens in patients receiving a high total dose of primaquine (7 mg/kg) was not possible since <4% of patients were treated with a 7-day high total dose regimen. Irrespective of the relative efficacy between 7-day and 14-day regimens in a trial setting, effectiveness will differ based on adherence,^34^ with shorter regimens more likely to have greater adherence and thus effectiveness.^35^

Single-dose radical cure with tafenoquine provides pragmatic advantages over prolonged daily primaquine regimens, which can be associated with poor adherence and low effectiveness.^34^, ^35^, ^36^ A large study in Brazil has demonstrated that tafenoquine is feasible and safe with high effectiveness.^11^^,^^37^ However, tafenoquine is currently only recommended for use in individuals with ≥70% G6PD activity, in combination with chloroquine and not with ACTs.^6^ Thus, despite a pro-active approach to implementing tafenoquine into clinical practice in Latin America,^8^^,^^11^ up to 30% of the population still rely on primaquine for radical cure of *P. vivax.* In settings where tafenoquine is contraindicated, unavailable, or operationally constrained, our findings highlight that high total-dose primaquine represents a viable, evidence-based alternative to improve radical cure.

We found no evidence that the daily dose of primaquine was associated with gastrointestinal intolerance or an increased risk of haemolysis. Our analyses were limited by a reduced number of studies reporting gastrointestinal symptoms and haematological safety data, and few patients were treated with high daily dose primaquine (≥0.75 mg/kg). However, the results of recent global meta-analyses assessing the effect of primaquine daily dose on tolerability and safety should be generalisable to Latin America.^9^^,^^10^ These analyses demonstrated a small increase in gastrointestinal symptoms with higher primaquine daily doses, but no increase in haemolytic risk in patients with ≥70% G6PD activity.^9^^,^^10^ Although in patients with intermediate deficiency (30 to ≤70% activity), high daily dose (1 mg/kg) primaquine may increase the risk of serious haemolysis,^38^ the absence of severe haemolytic events in the current analysis among individuals with ≥30% G6PD activity is reassuring. Our findings reinforce the importance of integrating G6PD testing into routine care to safely expand access to both optimised primaquine regimens and tafenoquine.

Our study has several additional limitations. Only a third of eligible studies contributed IPD, however, these studies were generally more recent and had extended follow-up. Incomplete reporting of gastrointestinal and haematological outcomes, particularly for higher daily-dose regimens, underscores the need for improved standardisation of safety monitoring in future trials. The primary outcome was *P. vivax* recurrences, which in addition to relapses can include reinfections and recrudescences (blood stage failure). The inability to directly assess relapses reflects the lack of a current standardised method to identify relapses in clinical trials from other causes of recurrence. Given relapses would be expected to occur at the same rate across all arms, their removal may increase the identified differences between treatment arms. Finally, only one included trial^29^ directly randomised high versus low total dose primaquine, which increases the potential for unmeasured confounding associated with individual study sites and thus strong causal conclusions cannot be drawn between primaquine dose and risk of recurrence.

In summary, our IPD meta-analysis provides regional evidence of the benefits of high total dose primaquine in reducing recurrent *P. vivax* in Latin America, and demonstrates that the regimen is safe when combined with G6PD testing in individuals in this region. Hence, high dose primaquine is a valuable alternative in patients in whom tafenoquine is contraindicated or not available. Our analysis also addresses the lack of certainty about primaquine dosing in Latin America, which was identified in the WHO Malaria Treatment Guidelines.^6^ Although few patients were included that received a 1 mg/kg daily primaquine dose, 0.5 mg/kg/day was well tolerated. As malaria programmes transition towards elimination, these findings directly inform policy decisions, supporting the strategic use of optimised primaquine regimens alongside tafenoquine, tailored to local epidemiology, health system capacity, and genetic risk profiles.

## Contributors

ADPC, MVGL, RNP, AMS, and RJC conceived the study. KN, ADPC, MR, HHMA, JAS, RNP, and RJC analysed and interpreted the data and drafted the manuscript. KN, RJC, and MR accessed and verified data. PJG provided technical support. ADPC, NNCS, AD, MUF, MSMG, LGC, JAG, GCKWK, MVGL, SL-A, ALC, WMM, SCN, AMDO, DBP, GMRV, JLFV, LMZ, and AMS conceived and undertook the individual studies and enrolled the patients. All the authors revised the manuscript. All authors have read and approved the final version of the manuscript. RJC was responsible for the decision to submit the manuscript.

## Data sharing statement

Pseudonymised participant data used in this analysis are available for access via the WWARN website (https://www.wwarn.org/). Request for access will be reviewed by data access committee to ensure the use of data protects the interests of the participants and researchers according to the terms of ethics approval and principles of equitable data sharing. Requests can be submitted by email to malariaDAC@iddo.org via the data access form available at https://www.wwarn.org/working-together/sharing-accessing-data/accessing-data is registered with the Registry of Research Data Repositories (https://www.re3data.org/). We acknowledge the inclusion of data from GlaxoSmithKline (GSK) made available through www.clinicalstudydatarequest.com.

## Declaration of interests

RJC and RNP report contributions to Up-To-Date. GCKWK reports holding shares within GSK and being a former employee of GSK. All other authors declare no competing interests.
