# Supplementary material for: A systematic review and individual patient data meta-analysis to determine the effect of primaquine dose on efficacy, tolerability and safety in uncomplicated Plasmodium vivax malaria in Latin America
Source: Lancet Reg Health Am. 2026 Sep 12;63:101634. doi: 10.1016/j.lana.2026.101634 (PMC13589060; doi:10.1016/j.lana.2026.101634)
Supplement: Supplementary file 1 [file mmc1.pdf]

# Appendix

Nguyen K, de Pina-Costa A, *et al*, A systematic review and individual patient data meta-analysis to determine the effect of primaquine dose on efficacy, tolerability and safety in uncomplicated *Plasmodium vivax* malaria in Latin America

## Table of Contents


Text S1. Supplementary methods ........ 3

Checklist S1. PRISMA-IPD Checklist of items to include when reporting a systematic review and meta-analysis of individual participant data (IPD) ........ 5

Figure S1. Timepoints of interest for efficacy, tolerability and safety analyses ........ 9

Figure S2. Directed acyclic graphs for the relationship between primaquine dose (exposure) and related outcomes. ........ 10

Table S1: Studies eligible for analyses but not included (23 studies) ........ 12

Table S2. Reasons for studies not being included in analyses ........ 15

Table S3: Studies included in analysis ........ 16

Table S4: Study testing and inclusion criteria ........ 17

Table S5: Study sites included in analysis ........ 18

Table S6. Comparison of baseline characteristics between included and eligible but not included studies in efficacy analysis ........ 19

Table S7: Risk of bias assessment in randomised controlled studies ........ 20

Tables S8: Risk of bias assessment in single arm observational studies ........ 21

Figure S3. Scatter plot of primaquine total dose (mg/kg) by A) age (years) and B) weight (kg) categorised by presence of recurrent parasitaemia between the day after the last primaquine dose to 150 days later ........ 22

Table S9. Sensitivity analyses for Cox regression analysis between total primaquine dose mg/kg and first recurrent *P. vivax* parasitaemia ........ 23

Table S10. Sensitivity analysis removing one study site at a time for relationship between total primaquine mg/kg dose and first recurrent *P. vivax* parasitaemia ........ 24

Figure S4. Cumulative incidence of first *P. vivax* recurrence in patients administered chloroquine by primaquine total dose ........ 25

Figure S5. Cumulative incidence of first *P. vivax* recurrence in patients administered chloroquine and a 14-day primaquine regimen by primaquine total dose ........ 26

Table S11. Dose groups, number of events and time at risk by study for the efficacy analysis assessing the cumulative incidence of *P. vivax* recurrence within 150 days of the last primaquine dose ........ 27

Figure S6. Two stage individual patient data meta-analysis comparing the hazard ratio for *P. vivax* recurrence following low total dose primaquine compared with no primaquine in studies randomising patients to these two arms ........ 28

Figure S7. Cumulative incidence of first *P. vivax* recurrence in patients administered chloroquine and low total dose primaquine by primaquine duration ........ 29

Table S12: Sensitivity analysis of cumulative incidence at day 150 excluding one study at a time in patients administered chloroquine and low total dose primaquine by regimen duration ........ 30

Figure S8. Cumulative incidence of first *P. vivax* recurrence in patients administered chloroquine and low total dose primaquine by primaquine duration, excluding Ladeia-Andrade 2019. ........ 31

Table S13. Demographic and baseline characteristics for patients included in the tolerability analysis ........ 32

Table S14: Demographic and baseline characteristics for patients with known G6PD activity included in the haematological safety analysis ........ 34

Figure S9. Aggregated data meta-analysis of trials comparing the risk of *P. vivax* recurrence following high versus low total dose primaquine undertaken in the Americas ........ 36

References S1 ........ 37

**Text S1. Supplementary methods**

***Systematic review***

All prospective *P. vivax* antimalarial clinical trials published between Jan 1, 2000 and July 3, 2026 were identified by the application of the key terms through Medline (Pubmed), Web of Science, Embase, Scopus and Cochrane Central. Abstracts of all references containing any mention of antimalarial drugs were manually checked to confirm prospective clinical trials, with review of full text when needed. This review was based on an original process that is documented in detail in Commons *et al*, Int J Parasitol Drug Drug Res 2017 and updated in Commons *et al*, Lancet Infect Dis 2024. The year of the study was taken as the year of publication, although the start and end dates of patient enrolment were also recorded.

The following search terms were used in Pubmed: vivax AND (artefenomel OR arterolane OR amodiaquine OR atovaquone OR artemisinin OR arteether OR artesunate OR artemether OR artemotil OR azithromycin OR artekin OR chloroquine OR chlorproguanil OR cycloguanil OR clindamycin OR coartem OR dapsone OR dihydroartemisinin OR duo-cotecxin OR doxycycline OR halofantrine OR lumefantrine OR lariam OR malarone OR mefloquine OR naphthoquine OR naphthoquinone OR piperaquine OR primaquine OR proguanil OR pyrimethamine OR pyronaridine OR proguanil OR quinidine OR quinine OR riamet OR sulphadoxine OR tetracycline OR tafenoquine); filtered for publication from Jan 1, 2000 to July 3, 2026. The systematic review was undertaken by two investigators (RJC and ADPC) with discrepancies resolved through discussion.

***Inclusion and exclusion criteria***

Studies were included in the analysis if:

- They were prospective clinical efficacy studies conducted in Latin America (all countries south of and including Mexico on the American continent)
- They had a minimum active follow-up of 28 days following presentation and start of antimalarial treatment
- All patients were treated with chloroquine or one of six common ACTs (artemether-lumefantrine, artesunate-mefloquine, artesunate-amodiaquine, dihydroartemisinin-piperaquine, artesunate-sulfadoxine-pyrimethamine, artesunate-pyronaridine)
- At least one treatment arm was included with daily primaquine administered over multiple days and commencing within 28 days of schizontocidal treatment.
- Individual patient data were available by July 26, 2024

Studies were excluded if data were from studies on prevention, prophylaxis, animal models, patients with severe malaria, studies where schizontocidal treatment was unsupervised (or in situations where it was dosed twice daily and both doses were unsupervised) or where data were extracted retrospectively outside of a planned trial were excluded.

Further inclusion criteria for efficacy analyses:

- Studies reporting parasite presence or absence during follow-up

- Patients followed for 42 days or more.

Further inclusion criteria for tolerability analyses:

- Studies in which patients were routinely asked about symptoms during follow-up using a checklist on gastrointestinal disturbance.

Further inclusion criteria for safety analyses:
- Patients with ≥30% G6PD activity
- Patients in whom haemoglobin or haematocrit were measured on the day primaquine was started and again during the next 14 days follow-up.

***Additional details on outcomes***

The primary efficacy outcome was any *P. vivax* recurrence (symptomatic or asymptomatic) occurring between the last dose of primaquine and 150 days after the last dose of primaquine. The endpoint of 150 days after the last dose of primaquine was chosen due to variable timing of primaquine administration between studies, which ranged from commencing on the day of presentation and start of schizontocidal treatment to up to 28 days later.[29] The 150-day endpoint was selected to maximise comparability across studies conducted in diverse transmission settings while capturing both early and intermediate relapse patterns.

***Additional analysis details***

Supervision of primaquine was classified as supervised (all doses directly observed), partially supervised (2 or more doses observed) and unsupervised (0 or 1 dose observed). Age groups were categorised as <5 years, 5-<15 and ≥15 years.

For time to event analyses, patients were left censored at the day after the last primaquine dose or the equivalent day for those who did not receive primaquine. Patients were right censored at time of the first episode of recurrent *P. vivax* parasitaemia, the first non-*P. vivax* parasitaemia, the day of last visit before loss to follow up, if there was more than a 67-day gap between blood smears, or the last day of the study, whichever occurred first.

*A priori* sensitivity analyses were undertaken for the primary efficacy outcome including restricting to patients treated with chloroquine as a partner drug, restricting to patients who received 14 dyas primaquine and were treated with chloroquine as a partner drug, restricting to patients commencing primaquine before day 7, restricting to patients where the actual primaquine dose was recorded, restricting to studies with 180 days or more follow up, analysis without shared frailty and removal of one study at a time and calculation of the coefficient of variation.

**Checklist S1. PRISMA-IPD Checklist of items to include when reporting a systematic review and meta-analysis of individual participant data (IPD)**

| PRISMA-IPD Section/topic | Item No | Checklist item | Reported on page |
|---|---|---|---|
| **Title** | | | |
| Title | 1 | Identify the report as a systematic review and meta-analysis of individual participant data. | 1 |
| **Abstract** | | | |
| Structured summary | 2 | Provide a structured summary including as applicable: | 5-6 |
| | | **Background**: state research question and main objectives, with information on participants, interventions, comparators and outcomes. | |
| | | **Methods**: report eligibility criteria; data sources including dates of last bibliographic search or elicitation, noting that IPD were sought; methods of assessing risk of bias. | |
| | | **Results**: provide number and type of studies and participants identified and number (%) obtained; summary effect estimates for main outcomes (benefits and harms) with confidence intervals and measures of statistical heterogeneity. Describe the direction and size of summary effects in terms meaningful to those who would put findings into practice. | |
| | | **Discussion:** state main strengths and limitations of the evidence, general interpretation of the results and any important implications. | |
| | | **Other:** report primary funding source, registration number and registry name for the systematic review and IPD meta-analysis. | |
| Introduction | | | |
| Rationale | 3 | Describe the rationale for the review in the context of what is already known. | 7-8 |
| Objectives | 4 | Provide an explicit statement of the questions being addressed with reference, as applicable, to participants, interventions, comparisons, outcomes and study design (PICOS). Include any hypotheses that relate to particular types of participant-level subgroups. | 7-8 |
| Methods | | | |
| Protocol and registration | 5 | Indicate if a protocol exists and where it can be accessed. If available, provide registration information including registration number and registry name. Provide publication details, if applicable. | 9 |
| Eligibility criteria | 6 | Specify inclusion and exclusion criteria including those relating to participants, interventions, comparisons, outcomes, study design and characteristics (e.g. years when conducted, required minimum follow-up). Note whether these were applied at the study or individual level i.e. whether eligible participants were included (and ineligible participants excluded) from a study that included a wider population than specified by the review inclusion criteria. The rationale for criteria should be stated. | 9-10, Appendix p3-4 |

| | | | |
|---|---|---|---|
| Identifying studies - information sources | 7 | Describe all methods of identifying published and unpublished studies including, as applicable: which bibliographic databases were searched with dates of coverage; details of any hand searching including of conference proceedings; use of study registers and agency or company databases; contact with the original research team and experts in the field; open adverts and surveys. Give the date of last search or elicitation. | 8-9, Appendix p3 |
| Identifying studies - search | 8 | Present the full electronic search strategy for at least one database, including any limits used, such that it could be repeated. | Appendix p3-4 |
| Study selection processes | 9 | State the process for determining which studies were eligible for inclusion. | 9, Appendix p3-4 |
| Data collection processes | 10 | Describe how IPD were requested, collected and managed, including any processes for querying and confirming data with investigators. If IPD were not sought from any eligible study, the reason for this should be stated (for each such study). | 9 |
| | | If applicable, describe how any studies for which IPD were not available were dealt with. This should include whether, how and what aggregate data were sought or extracted from study reports and publications (such as extracting data independently in duplicate) and any processes for obtaining and confirming these data with investigators. | Appendix p12-15 |
| Data items | 11 | Describe how the information and variables to be collected were chosen. List and define all study level and participant level data that were sought, including baseline and follow-up information. If applicable, describe methods of standardising or translating variables within the IPD datasets to ensure common scales or measurements across studies. | Appendix p3-4, ref 14 |
| IPD integrity | A1 | Describe what aspects of IPD were subject to data checking (such as sequence generation, data consistency and completeness, baseline imbalance) and how this was done. | Ref 14 |
| Risk of bias assessment in individual studies. | 12 | Describe methods used to assess risk of bias in the individual studies and whether this was applied separately for each outcome. If applicable, describe how findings of IPD checking were used to inform the assessment. Report if and how risk of bias assessment was used in any data synthesis. | 13, Appendix p20-21 |
| Specification of outcomes and effect measures | 13 | State all treatment comparisons of interests. State all outcomes addressed and define them in detail. State whether they were pre-specified for the review and, if applicable, whether they were primary/main or secondary/additional outcomes. Give the principal measures of effect (such as risk ratio, hazard ratio, difference in means) used for each outcome. | 11-13, Appendix p4 |
| Synthesis methods | 14 | Describe the meta-analysis methods used to synthesise IPD. Specify any statistical methods and models used. Issues should include (but are not restricted to):<br>• Use of a one-stage or two-stage approach.<br>• How effect estimates were generated separately within each study and combined across studies (where applicable).<br>• Specification of one-stage models (where applicable) including how clustering of patients within studies was accounted for.<br>• Use of fixed or random effects models and any other model assumptions, such as proportional hazards. | 11-13 |

| | | | |
|---|---|---|---|
| | | • How (summary) survival curves were generated (where applicable).<br>• Methods for quantifying statistical heterogeneity (such as $I^2$ and $\tau^2$).<br>• How studies providing IPD and not providing IPD were analysed together (where applicable).<br>• How missing data within the IPD were dealt with (where applicable). | |
| Exploration of variation in effects | A2 | If applicable, describe any methods used to explore variation in effects by study or participant level characteristics (such as estimation of interactions between effect and covariates). State all participant-level characteristics that were analysed as potential effect modifiers, and whether these were pre-specified. | 12, Appendix p10-11 |
| Risk of bias across studies | 15 | Specify any assessment of risk of bias relating to the accumulated body of evidence, including any pertaining to not obtaining IPD for particular studies, outcomes or other variables. | 13 |
| Additional analyses | 16 | Describe methods of any additional analyses, including sensitivity analyses. State which of these were pre-specified. | 15-16, Appendix p4 |
| Results | | | |
| Study selection and IPD obtained | 17 | Give numbers of studies screened, assessed for eligibility, and included in the systematic review with reasons for exclusions at each stage. Indicate the number of studies and participants for which IPD were sought and for which IPD were obtained. For those studies where IPD were not available, give the numbers of studies and participants for which aggregate data were available. Report reasons for non-availability of IPD. Include a flow diagram. | 14, Figure 1 |
| Study characteristics | 18 | For each study, present information on key study and participant characteristics (such as description of interventions, numbers of participants, demographic data, unavailability of outcomes, funding source, and if applicable duration of follow-up). Provide (main) citations for each study. Where applicable, also report similar study characteristics for any studies not providing IPD. | Appendix 16-19 |
| IPD integrity | A3 | Report any important issues identified in checking IPD or state that there were none. | **Ref 14** |
| Risk of bias within studies | 19 | Present data on risk of bias assessments. If applicable, describe whether data checking led to the up-weighting or down-weighting of these assessments. Consider how any potential bias impacts on the robustness of meta-analysis conclusions. | 14, Appendix p20-21 |
| Results of individual studies | 20 | For each comparison and for each main outcome (benefit or harm), for each individual study report the number of eligible participants for which data were obtained and show simple summary data for each intervention group (including, where applicable, the number of events), effect estimates and confidence intervals. These may be tabulated or included on a forest plot. | Appendix p16, 28 |
| Results of syntheses | 21 | Present summary effects for each meta-analysis undertaken, including confidence intervals and measures of statistical heterogeneity. State whether the analysis was pre-specified, and report the numbers of studies and participants and, where applicable, the number of events on which it is based. | 14-18 |
| | | When exploring variation in effects due to patient or study characteristics, present summary interaction estimates for each characteristic examined, including confidence intervals and measures of statistical heterogeneity. State whether the analysis was pre-specified. State whether any interaction is consistent across trials. | |
| | | Provide a description of the direction and size of effect in terms meaningful to those who would put findings into practice. | |

| | | | |
|---|---|---|---|
| Risk of bias across studies | 22 | Present results of any assessment of risk of bias relating to the accumulated body of evidence, including any pertaining to the availability and representativeness of available studies, outcomes or other variables. | 14, Appendix p19 |
| Additional analyses | 23 | Give results of any additional analyses (e.g. sensitivity analyses). If applicable, this should also include any analyses that incorporate aggregate data for studies that do not have IPD. If applicable, summarise the main meta-analysis results following the inclusion or exclusion of studies for which IPD were not available. | 15-16, Appendix p23-31 |
| **Discussion** | | | |
| Summary of evidence | 24 | Summarise the main findings, including the strength of evidence for each main outcome. | 18-23 |
| Strengths and limitations | 25 | Discuss any important strengths and limitations of the evidence including the benefits of access to IPD and any limitations arising from IPD that were not available. | 22-23 |
| Conclusions | 26 | Provide a general interpretation of the findings in the context of other evidence. | 23 |
| Implications | A4 | Consider relevance to key groups (such as policy makers, service providers and service users). Consider implications for future research. | **23** |
| **Funding** | | | |
| Funding | 27 | Describe sources of funding and other support (such as supply of IPD), and the role in the systematic review of those providing such support. | 25-26 |

© Reproduced with permission of the PRISMA IPD Group, which encourages sharing and reuse for non-commercial purposes

**Figure S1. Timepoints of interest for efficacy, tolerability and safety analyses**

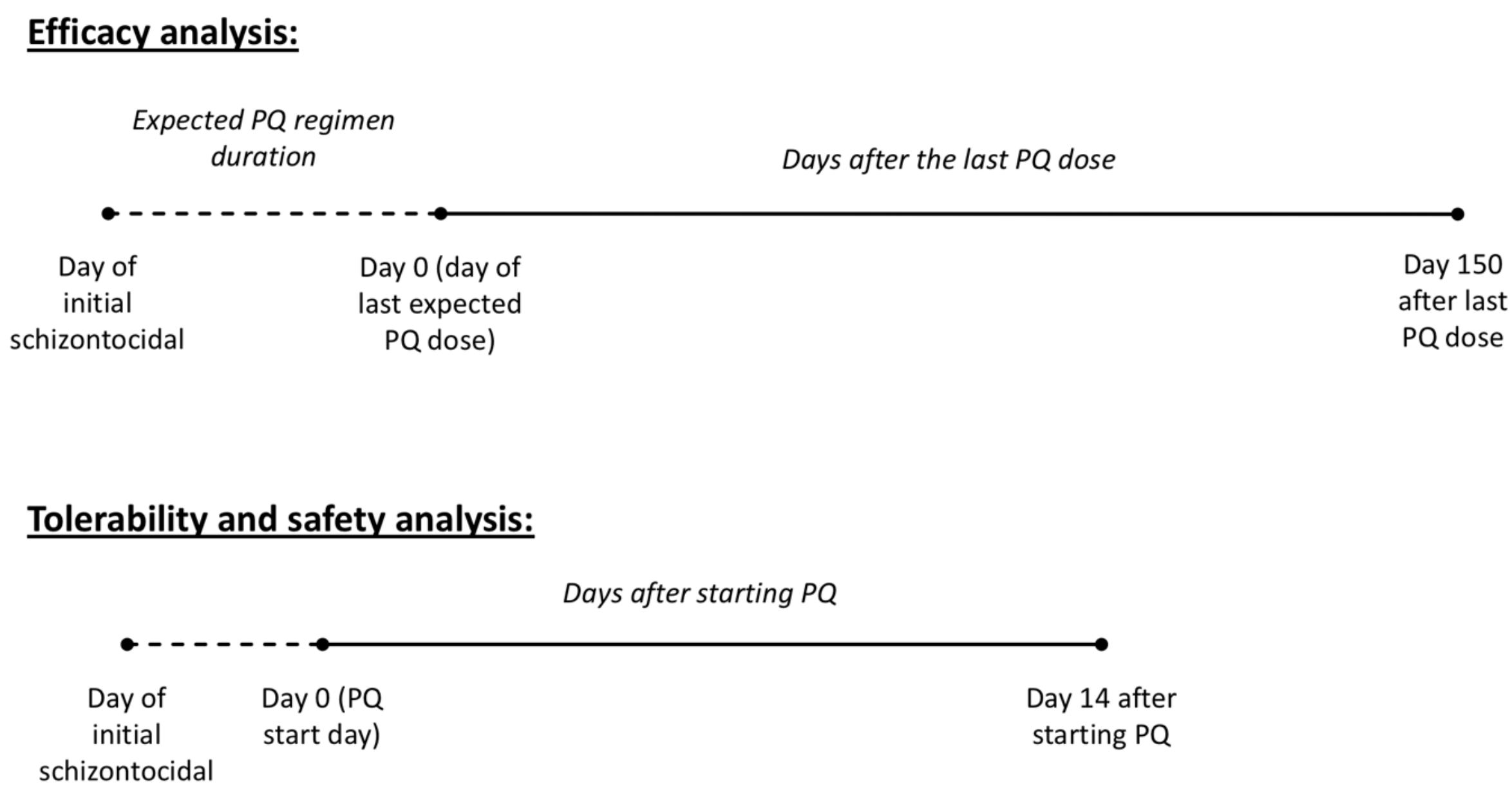

**Figure S2. Directed acyclic graphs for the relationship between primaquine dose (exposure) and related outcomes.**

A. The relationship between total primaquine mg/kg dose and time to first *P.vivax* recurrence

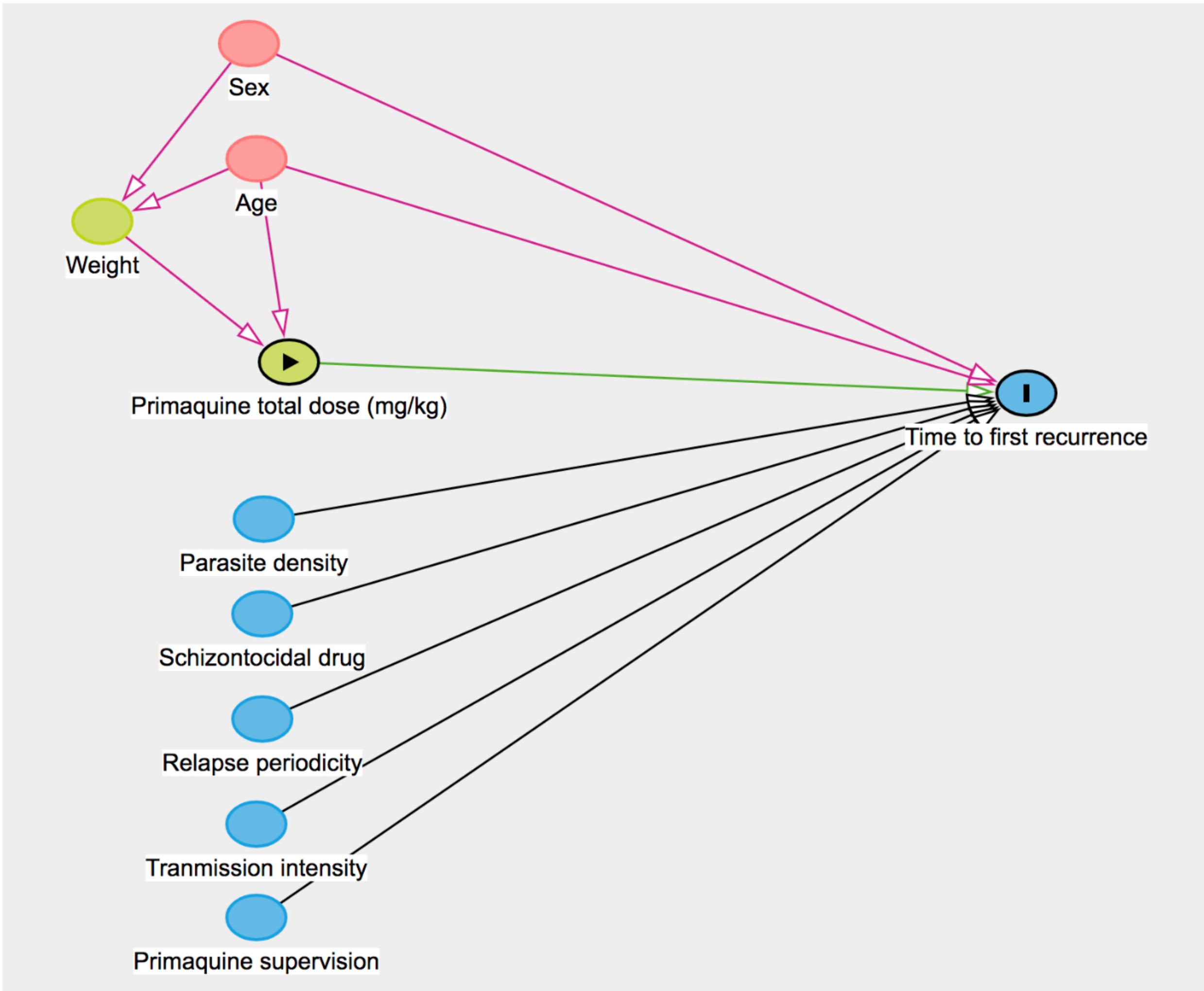

B. The relationship between daily primaquine mg/kg dose and gastrointestinal disturbance

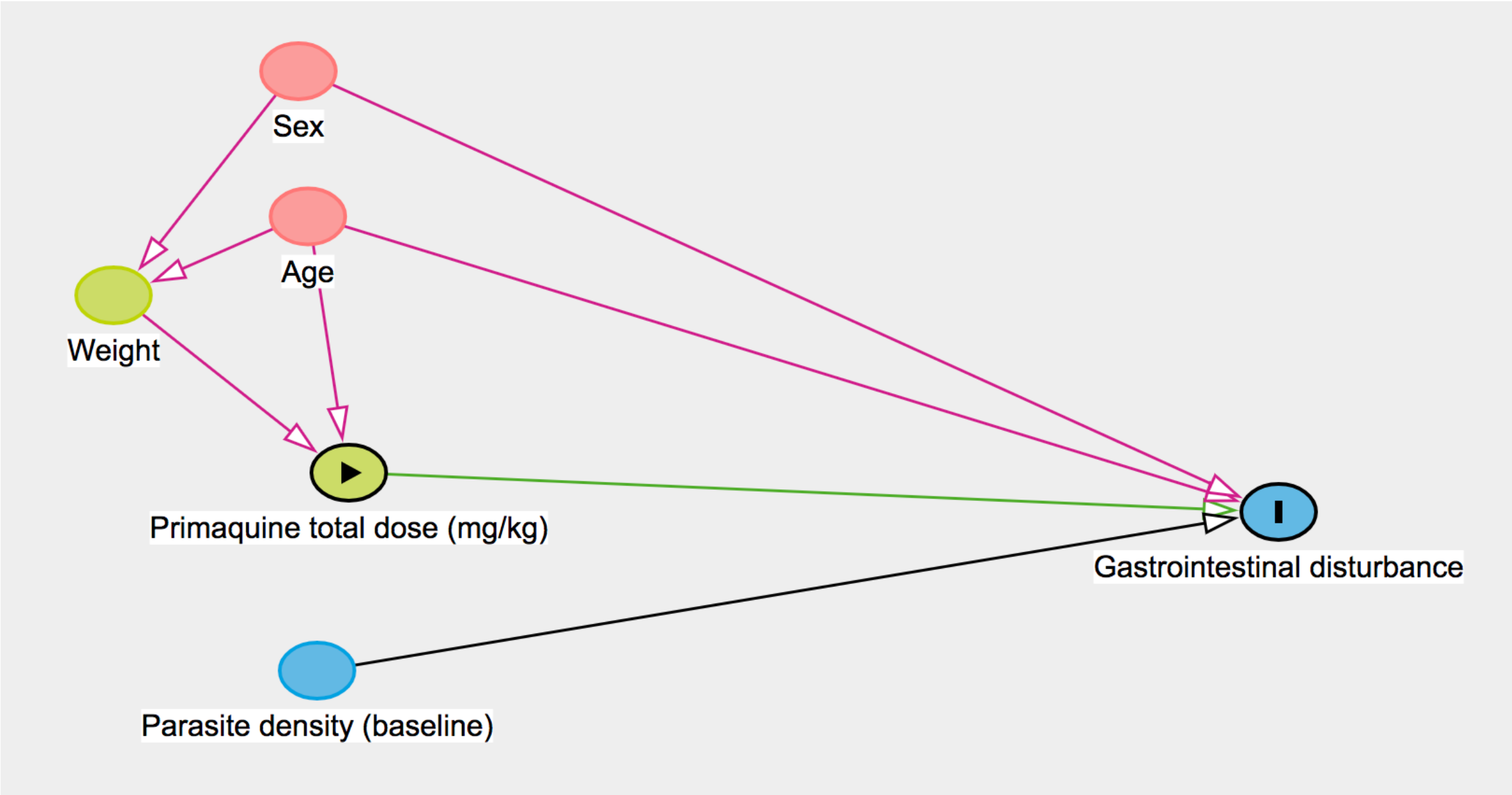


C. The relationship between daily primaquine mg/kg dose and haemoglobin

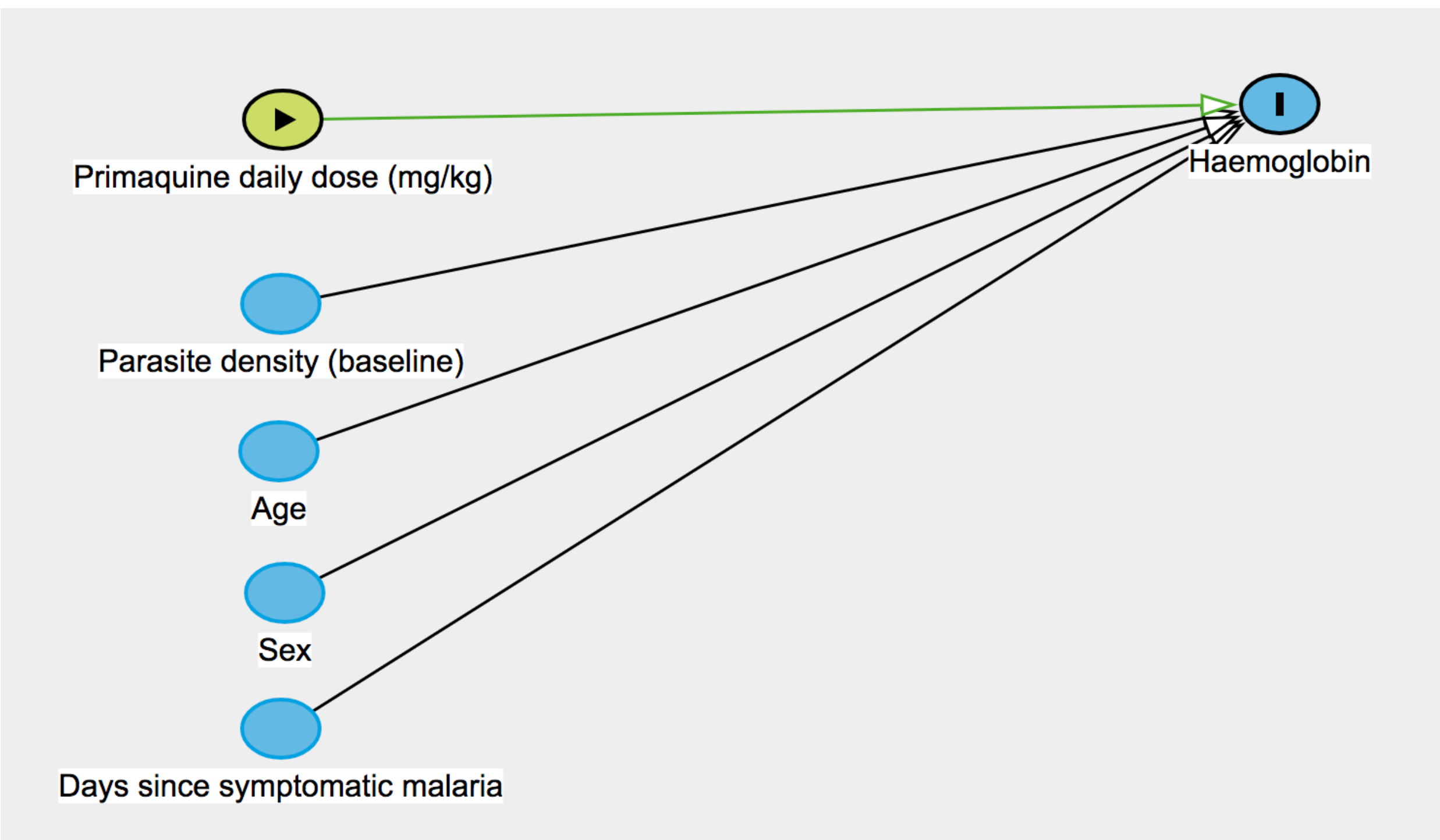

## Table S1: Studies eligible for analyses but not included (23 studies)

| First Author | Treatment groups | Country | Follow up (days) | Randomised | Recruitment period | Treatment groups | Pv patients enrolled | Treated with PQ | Female (%) | Mean Age (SD) | Median Age (range) | Reasons for exclusion | Eligibility | | |
|---|---|---|---|---|---|---|---|---|---|---|---|---|---|---|---|
| | | | | | | | | | | | | | Efficacy | GI tol | Haem |
| Bergonzoli-2000[39] | 4 | Costa Rica | 180 | Yes | 1994 | Cq_Pq_3.5_14d_D0; Cq_Pq_2.75_9d_D0; Cq_Pq_2.0_5d_D0; Cq_Pq_0.75_1d_D0 | 132 | 132 | Not stated | 30.5 (-) | | Unable to contact | Yes | Yes | No |
| Villalobos-Salcedo-2000[40] | 2 | Brazil | 28 | Yes | 1998 | Cq_Pq_3.5_14d_D3; Cq_Pq_2.5_5d_D0 | 79 | 79 | 21.5 | 31.7 (-) | | No response | No | No | No |
| Abdon-2001[41] | 3 | Brazil | 180 | Yes | 1994-1995 | Cq_Pq_3.5_14d_D0; Cq_Pq_3.5_7d_D0; Cq_Pq_2.5_5d_D0 | 120 | 120 | 37.5 | 27.3 (-) | | No response | Yes | No | No |
| Duarte-2001[42] | 1 | Brazil | 180 | No | 1997-1998 | Cq_Pq_3.5_14d_D2 | 50 | 50 | 24.0 | 31.8 (12.8) | | No response | Yes | Yes | No |
| Solari Soto-2002[43] | 2 | Peru | 60 | Yes | 1998-1999 | Cq_Pq_3.5_7d_D2; Cq_Pq_3.5_14d_D2 | 60 | 60 | 43.3 | 26.45 (16.96) | | No response | Yes | Yes | No |
| da Silva-2003[44] | 8 | Brazil | 180 | Yes | Not stated | Cq_Pq_3.5_7d_D2; Cq_Pq_2.5_5d_D2; As_Pq_3.5_7d_D2; As_Pq_3.5_7d_D2; As_Pq_3.5_7d_D2; As_Pq_2.5_5d_D2; As_Pq_2.5_5d_D2; As_Pq_2.5_5d_D2 | 240 | 240 | 23.3 | 32.9 (-) | | Unable to contact | Yes | Yes | No |
| Machado-2003[45] | 1 | Brazil | 30 | No | N/A | Cq_Pq_3.5_14d_D3 | 30 | 30 | Not stated | Not stated | | No response | Yes | Yes | No |
| Pinto-2003[46] | 2 | Brazil | 28 | Yes | 1997-1998 | Cq_Pq_3.0_14d_D0; Cq_Pq_3.0_14d_D0 | 132 | 132 | 37.9 | 30.7 (-) | | No response | No | Not stated | No |
| Alvarez-2006[47] | 3 | Colombia | 180 | Yes | 2001 | Cq_Pq_0.75_3d_D1; Cq_Pq_1.75_7d_D1; Cq_Pq_3.5_14d_D1 | 210 | 210 | 33.0 | 30.1 (12.8) | | Missing minimum data | Yes | No | No |
| Carmona-Fonseca-2006[48] | 3 | Colombia | 28 | Yes | 2003-2004 | Cq_Pq_0.8_3d_D0; Cq_Pq_1.8_7d_D0; Cq_Pq_3.5_14d_D0; | 228 | 228 | 32.0 | 30.1 (-) | | Data not available | Yes | Yes | No |
| Perez-2008[49] | 1 | Colombia | 28 | No | 2006 | Cq_Pq_4.2_14d_D7 | 50 | 50 | 18.5 | Not stated | | No response | Yes | Yes | No |

| | | | | | | | | | | | | | | | |
|---|---|---|---|---|---|---|---|---|---|---|---|---|---|---|---|
| Carmona-Fonseca-2009[50] | 4 | Colombia | 120 | Yes | 2001-2003 | Cq_Pq_3.5_14d_D1; Cq_Pq_1.75_3d_D1; Cq_Pq_2.5_3d_D1; Cq_Pq_3.5_3d_D1 | 188 | 188 | 30.4 | Not stated | | Data not available | Yes | No | No |
| Orjuela-Sanchez-2009[51] | 2 | Brazil | 336 | No | 2004-2007 | Cq_Pq_3.5_7d_D0; Cq_Pq_3.5_7d_D0 | 164 | 164 | Not stated | Not stated | | Missing minimum data | Yes | No | No |
| Carmona-Fonseca-2010[52] | 2 | Colombia | 120 | Yes | 2005-2008 | Cq_Pq_3.5_7d_D1; Cq_Pq_3.5_3d_D1 | 79 | 79 | Not stated | Not stated | | Data not available | Yes | Yes | No |
| Van Den Eede-2011[53] | 1 | Peru | 365 | No | 2008 | Cq_Pq_3.5_7d_D0 | 51 | 51 | 49.0 | Not stated | 15 (2-80) | Data not provided | Yes | Yes | No |
| Eibach-2012[54] | 1 | Guyana | 28 | No | 2009-2010 | Al_Pq_3.5_14d_D0 | 74 | 74 | 9.5 | Not stated | 24 (5-57) | Unable to contact | No | No | No |
| Graf-2012[55] | 3 | Peru | 210 | Yes | 2005-2008 | Cq_Pq_2.5_5d_D0; Cq_Pq_3.5_7d_D0; Cq_Pq_3.5_14d_D0 | 540 | 540 | Not stated | Not stated | | No response | Yes | No | No |
| Pedro-2012[56] | 1 | Brazil | 28 | No | 2005-2011 | Cq_Pq_3.5_Xd_D0 | 47 | 47 | 24.5 | Not stated | | No response | No | No | No |
| Delgado-Ratto-2014[57] | 1 | Peru | 720 | No | 2008 | Cq_Pq_3.5_7d_D0 | 37 | 37 | 48.6 | Not stated | 15 | No response | Yes | Yes | No |
| Negreiros-2016[58] | 1 | Brazil | 168 | No | 2014 | Cq_Pq_3.5_7d_D0 | 119 | 119 | 45.4 | Not stated | 23.4 (5-67.3) | Data not provided | Yes | Yes | No |
| Mac Donald-Ottevanger-2018[59] | 2 | Suriname | 365 | Yes | 2006-2008 | Cq_Pq_3.5_7d_D3; Cq_Pq_3.5_14d_D3 | 79 | 79 | 34.4 | Not stated | 24.64 (-) | No response | Yes | No | No |
| Brasil-2018[60] | 1 | Brazil | 180 | No | 2012-2014 | Cq_Pq_3.5_7d_D0 | 213 | 213 | 43.4 | Not stated | | Missing minimum data | Yes | No | No |
| Mesa-Echeverry-2019[61] | 1 | Colombia | 28 | No | 2012-2013 | Cq_Pq_3.5_14d_D0 | 80 | 80 | 48.0 | 25.8 (14.4) | 22 (-) | No response | Yes | Yes | No |
| Barbosa-2025[62] | 4 | Brazil | 180 | Yes | 2018-2022 | Cq_Pq_3.5_7d_D4; Cq_Pq_6.0_8w_D0; Cq_12w; Cq_Pq_3.5_7d_D0 | 106 | 80 | 16.0 | 38.9 (-) | | Not available by July 26, 2024 | Yes | No | Yes |
| Calderon-Ruiz-2024[63] | 4 | Colombia | 28 | No | 2020-2022 | Cq_Pq_3.5_7d_D0_sup; Cq_Pq_3.5_7d_D0_unsup ; Cq_Pq_3.5_14d_D0_sup; | 103 | 103 | 39.8 | Not stated | | Not available by July 26, 2024 | Yes | No | No |

| | | | | | | | | | | | | | | | |
|---|---|---|---|---|---|---|---|---|---|---|---|---|---|---|---|
| | | | | | | Cq_Pq_3.5_14d_D0_ unsup | | | | | | | | | |
| Pacheco-2025[31] | 3 | Brazil | 180 | Yes | 2021-2025 | Cq_Pq_3.5_7d_D0; Cq_Pq_7.0_14d_D0; Cq_Pq_7.0_7d_D0 | 100 | 100 | 45 | Not stated | 10.1 (6.0-12.0) | Not available by July 26, 2024 | Yes | Yes | Yes |
| Godinho-2026[64] | 2 | Brazil | 180 | No | 2022-2023 | Cq_Pq_3.5_7d_D0; Cq_Tq | 118 | 67 | 26.4 | Not stated | 42.5 (32.0-52.0) | Not available by July 26, 2024 | Yes | No | No |

Al – artemether-lumefantrine; As – artesunate; Cq – chloroquine; PQ/Pq – primaquine; Pv – *P. vivax*; SD – standard deviation; sup – supervised; unsup – unsupervised;*Treatment code describes (schizontocidal drug)_(hypnozoitocidal drug)_(total primaquine dose)_(duration of primaquine treatment eg 14d = 14 days)_(primaquine start day)

**Table S2. Reasons for studies not being included in analyses**

| Reason | Number of studies | Studies |
|---|---|---|
| Data not available | 3 | [48,50,52] |
| Investigators unable to be contacted | 3 | [39,44,54] |
| Missing minimum data for inclusion | 3 | [47,51,60] |
| No data available by July 26, 2024 | 2 | [31,53,58,62-64] |
| No response from investigators | 12 | [40-43,45,46,49,55-57,59,61] |

**Table S3: Studies included in analysis**

| Author-year | Country | Recruitment Period | Age range (years) | Follow up (days) | Included treatment groups* | PQ supervision | Randomised | Patients available | Included in GI tolerability analysis | Included in haematology analysis | Included in efficacy analysis |
|---|---|---|---|---|---|---|---|---|---|---|---|
| Llanos-Cuentas-2014[18] | Multinational | 2010-2013 | 16-72 | 180 | Cq, Cq_Pq_3.5_14d_D1 | Partial | Yes | 224 | No | Yes | Yes |
| Marques-2014[19] | Brazil | 2007 – 2008 | 13-65 | 28 | Cq_Pq_3.5_7-9d_D0 | Full | No | 154 | No | No | No |
| Gomes-2015[20] | Brazil | 2011 | 10-56 | 28 | Cq_Pq_3.5_7-12d_D0 | Full | No | 94 | No | No | No |
| Gonzalez-Ceron-2015[21] | Mexico | 2008 - 2010 | 3-78 | 365 | Cq_Pq_3.5_14d_D0 | Full | Yes | 159 | No | No | Yes |
| Pereira-2016[22] | Brazil | 2013 - 2014 | 19-68 | 28 | Cq_Pq_3.5_7-9d_D0 | Partial | No | 88 | Yes | No | No |
| Zuluaga-Idarraga-2016[23] | Colombia | 2012 - 2013 | 4-71 | 180 | Cq_Pq_3.5_14d_D0 | Full | No | 87 | Yes | No | Yes |
| Daher-2018[24] | Brazil | 2012 - 2015 | 18-65 | 63 | Al_Pq_3.5_7-9d_D0, AsMf_Pq_3.5_7-9d_D0, Cq_Pq_3.5_7-9d_D0 | Partial | Yes | 264 | No | No | Yes |
| de Sena-2019[25] | Brazil | 2016 - 2017 | 2-14 | 42 | Cq_Pq_3.5_7d_D0 | Partial | No | 113 | No | No | Yes |
| Lacerda- 2019[26] | Multinational | 2013-2017 | 15-71 | 180 | Cq, Cq_Pq_3.5_14d_D1 | Partial | Yes | 478 | No | Yes | Yes |
| Ladeia-Andrade-2019[27] | Brazil | 2014 - 2015 | 7-60 | 180 | Cq_Pq_3.5_7d_D0 | Full | Yes | 204 | Yes | Yes | Yes |
| Llanos-Cuentas-2019[28] | Multinational | 2014 - 2017 | 15-74 | 180 | Cq_Pq_3.5_14d_D1 | Partial | No | 265 | No | No | Yes |
| Chamma-Siqueira-2022[29] | Brazil | 2018 - 2019 | 5.4-79.8 | 168 | Cq_Pq_3.5_7d_D17_obs, Cq_Pq_3.5_7d_D17_unobs, Cq_Pq_7.0_14d_D17 | Both (Full and No) | Yes | 254 | No | Yes | Yes |
| Baia-da-Silva-2026[30] | Brazil | 2018-2021 | 9-84 | 180 | Cq_Pq_7.0_14d_D0, Cq_Pq_7.0_14d_D42, Dp_Pq_7.0_14d_D0, Dp_Pq_7.0_14d_D42 | Partial | Yes | 419 | Yes | Yes | Yes |

Al – artemether-lumefantrine; As – artesunate; Cq – chloroquine; Dp – dihydroartemisinin-piperaquine; GI – gastrointestinal; Mf – mefloquine; PQ/Pq – primaquine; *Treatment code describes (schizontocidal drug)_(hypnozoitocidal drug)_(total primaquine dose)_(duration of primaquine treatment eg 14d = 14 days)_(primaquine start day)

## Table S4: Study testing and inclusion criteria

| Author-year | Hb exclusion criteria | G6PD testing undertaken | G6PD activity included | G6PD data available | Days symptom checklist undertaken and data available | Days Hb/Hct measured and data available |
|---|---|---|---|---|---|---|
| Llanos-Cuentas-2014[18] | Hb <7g/dL | Spectrophotometry | ≥70% activity | Yes | 0 | 0, 2, 3, 4, 5, 6, 7, 8, 9, 10, 11, 12 ,13, 14 |
| Marques-2014[19] | No exclusions | Not stated | Unknown | No | - | 0, 3, 7, 14 |
| Gomes-2015[20] | Not stated | Not stated | Unknown | No | - | 0 |
| Gonzalez-Ceron-2015[21] | Not stated | No | All activities | No | 0, 2, 3, 7 | - |
| Pereira- 2016[22] | Not stated | No | ≥30% activity | No | 0, 1, 2, 3, 7, 14, 21, 28 | 0, 14, 28 |
| Zuluaga-Idarraga-2016[23] | Hb <5 g/dL | No | All activities | No | 0, 1, 2, 3, 7, 13 | - |
| Daher- 2018[24] | Hb <7g/dL | No | All activities | No | - | 0, 14, 28, 42, 63 |
| de Sena-2019[25] | Hb <5 g/dL | Not stated | Unknown | No | - | 0 |
| Lacerda- 2019[26] | Hb <7g/dL | Spectrophotometry | ≥70% activity | Yes | 0 | 0, 1, 2, 3, 4, 5, 6, 7, 8, 9, 10, 11, 12 ,13, 14 |
| Ladeia-Andrade-2019[27] | Hb <8 g/dL | BinaxNow RDT | ≥30% activity | Yes | 0, 1, 2, 3, 7, 14 | 0, 1, 2, 3, 7, 14 |
| Llanos-Cuentas- 2019[28] | Hb <7g/dL | Spectrophotometry | ≥70% activity | Yes | 0 | 0, 2, 3, 4, 5, 6, 7, 8, 9, 10, 11, 12 ,13, 14 |
| Chamma-Siqueira-2022[29] | Not stated | Yes | ≥7 U/gram Hb | Yes | 0 | 0, 14, 28 |
| Baia-da-Silva-2026[30] | Hb <7g/dL | Yes | ≥4.0 U/gram Hb (SD Biosensor) | No | 0 | 0, 6, 13, 20, 27 |

**Table S5: Study sites included in analysis**

| Paper | Study site | Country | Region | Lat | Long | Year Start | Year End | MAP Incidence rate (per 1000 persons) | Transmission intensity* | Relapse periodicity# |
|---|---|---|---|---|---|---|---|---|---|---|
| Llanos-Cuentas-2014[18] | Manaus | Brazil | Americas | -3.12 | -60.02 | 2011 | 2013 | 42.81 | High | Low |
| Marques-2014[19] | Manaus | Brazil | Americas | -3.11 | -60.03 | 2007 | 2008 | 73.17 | High | Low |
| Gomes-2015[20] | Oiapoque-AP | Brazil | Americas | 3.84 | -51.83 | 2011 | 2011 | 37.70 | High | Low |
| Gonzalez-Ceron-2015[21] | Huehuetan | Mexico | Americas | 15.06 | -92.33 | 2008 | 2009 | 0.55 | Low | Low |
| Gonzalez-Ceron-2015[21] | Tuxtla Chico | Mexico | Americas | 14.91 | -92.15 | 2008 | 2009 | 0.55 | Low | Low |
| Gonzalez-Ceron-2015[21] | Fr Hidalgo | Mexico | Americas | 14.78 | -92.18 | 2008 | 2009 | 0.55 | Low | Low |
| Gonzalez-Ceron-2015[21] | Cacahoatan | Mexico | Americas | 15.09 | -92.21 | 2008 | 2009 | 0.55 | Low | Low |
| Gonzalez-Ceron-2015[21] | Tapachula | Mexico | Americas | 14.91 | -92.26 | 2008 | 2009 | 0.55 | Low | Low |
| Pereira-2016[22] | Porto Velho | Brazil | Americas | -11.51 | -63.58 | 2013 | 2015 | 11.50 | High | Low |
| Zuluaga-Idarraga-2016[23] | Turbo | Colombia | Americas | 8.10 | -76.73 | 2012 | 2012 | 5.08 | Moderate | Low |
| Daher-2018[24] | Porto Velho | Brazil | Americas | -11.51 | -63.58 | 2013 | 2014 | 11.50 | High | Low |
| Daher-2018[24] | Manaus | Brazil | Americas | -3.12 | -60.02 | 2013 | 2014 | 30.81 | High | Low |
| de Sena-2019[25] | Marajo Island | Brazil | Americas | -0.94 | -49.64 | 2016 | 2017 | 5.89 | Moderate | Low |
| Lacerda-2019[26] | Manaus | Brazil | Americas | -3.12 | -60.02 | 2013 | 2016 | 41.14 | High | Low |
| Ladeia-Andrade-2019[27] | Mancio Lima | Brazil | Americas | -7.61 | -72.91 | 2014 | 2014 | 47.40 | High | Low |
| Llanos-Cuentas-2019[28] | Cali | Colombia | Americas | 3.45 | -76.53 | 2015 | 2016 | 1.93 | Moderate | Low |
| Llanos-Cuentas-2019[28] | Manaus | Brazil | Americas | -3.12 | -60.02 | 2015 | 2016 | 18.55 | High | Low |
| Llanos-Cuentas-2019[28] | Iquitos | Peru | Americas | -3.74 | -73.25 | 2015 | 2016 | 58.05 | High | Low |
| Chamma-Siqueira-2022[29] | Cruzeiro do Sul | Brazil | Americas | -7.63 | -72.68 | 2018 | 2019 | 19.97 | High | Low |
| Baia-da-Silva-2026[30] | Manaus | Brazil | Americas | -3.12 | -60.02 | 2018 | 2021 | 25.92 | High | Low |

Lat – latitude; Long – longitude; MAP – Malaria Atlas Project; *Transmission intensity is classified as low (an incidence rate of <1 per 1000 persons), moderate (1 to <10 per 1000 persons), high (≥10 per 1000 persons); # High relapse periodicity <47 days.

**Table S6. Comparison of baseline characteristics between included and eligible but not included studies in efficacy analysis**

| Characteristic | Included studies (n=10) | Eligible but not included (n=27) |
|---|---|---|
| *Countries included, studies (%)[#]* | | |
| Brazil | 6.3 (63%) | 13 (48%) |
| Colombia | 1.3 (13%) | 7 (26%) |
| Mexico | 1 (10%) | - |
| Peru | 1.3 (13%) | 4 (15%) |
| Costa Rica | - | 1 (4%) |
| Guyana | - | 1 (4%) |
| Suriname | - | 1 (4%) |
| *Year of enrolment, studies (%)†* | | |
| Pre 2009 | 1 (10%) | 16 (64%) ‡ |
| Post 2009 | 9 (90%) | 9 (36%) ‡ |
| *Follow up duration (days)* | | |
| ≤42 | 1 (10%) | 9 (33%) |
| >42 to <180 | 2 (20%) | 4 (15%) |
| 180 | 6 (60%) | 9 (33%) |
| >180 | 1 (10%) | 5 (19%) |
| Age (years), median (IQR) | 32 (19-45) | 30 (26-32) § |
| Female, % of patients, median (IQR) | 35.3 ¶ | 33 (25-43) ^ |

IQR- interquartile range; [#]Multinational studies are considered a proportion of the number of studies within each country. *Age and female percentage of targeted studies calculated using frequency weighted mean or median according to number of patients. † Year of enrolment defined as year study enrolment completed. ‡ Year of enrolment not available for categorisation from 2 studies. § Mean/median age not available from 9 studies. ¶ No IQR presented as actual percentage of females included in studies. ^Number of female patients unavailable in 5 studies.

**Table S7: Risk of bias assessment in randomised controlled studies**

| Author-year | Bias from randomisation | Bias due to deviation from intervention | Bias from missing outcome | | | Bias in measurement of the outcome | | | Bias in selection of the reported results | Overall bias | Follow up to 180 days | Comparison of no PQ to PQ |
|---|---|---|---|---|---|---|---|---|---|---|---|---|
| | | | Efficacy | Haematology | Tolerability | Efficacy | Haematology | Tolerability | | | | |
| Llanos-Cuentas-2014[18] | Green | Green | Green | Green | Grey | Green | Green | Grey | Green | Green | Green | Green |
| Gonzalez-Ceron-2015[21] | Red | Green * | Orange | Grey | Green | Green | Grey | Orange | Orange | Red | Green | Red |
| Daher-2018[24] | Green | Green | Orange | Green | Grey | Green | Green | Grey | Green | Orange | Red | Red |
| Lacerda-2019[26] | Green | Green | Green | Green | Grey | Green | Green | Grey | Green | Green | Green | Green |
| Ladeia-Andrade-2019[27] | Green | Green | Orange | Green | Green | Green | Green | Orange | Orange | Orange | Green | Red |
| Llanos-Cuentas-2019[28] | Green | Green | Green | Green | Green | Green | Green | Green | Green | Green | Green | Green |
| Chamma-Siqueira-2022[29] | Green | Green | Green | Green | Green | Green | Green | Green | Green | Green | Red | Red |
| Baia-da-Silva-2026[30] | Green | Green | Green | Green | Green | Green | Green | Green | Green | Green | Green | Red |

Green – low risk of bias; Red – high risk of bias; Orange – unclear risk of bias; Grey – not applicable; Assessed according to the Cochrane Risk of Bias 2 tool[16] for randomised controlled trials; * Studies analysed per protocol but all data available for these meta-analyses; PQ – primaquine.

## Tables S8: Risk of bias assessment in single arm observational studies

| Author-year | Clear criteria for inclusion | Condition measured in reliable way | Valid methods for condition | Consecutive inclusion | Complete inclusion | Demographics reported | Clinical information reported | Outcomes reported | Site description | Analysis appropriate | Follow up to 180 days | Comparison of no PQ to PQ |
|---|---|---|---|---|---|---|---|---|---|---|---|---|
| Marques-2014[19] | Green | Green | Green | Red | Grey | Green | Green | Green | Green | Green | Grey | Grey |
| Gomes-2015[20] | Green | Green | Green | Green | Green | Green | Green | Green | Green | Green | Grey | Grey |
| Pereira- 2016[22] | Green | Green | Green | Green | Green | Green | Green | Green | Green | Green | Red | Grey |
| Zuluaga-Idarraga-2016[23] | Green | Green | Green | Green | Green | Green | Green | Green | Green | Green | Green | Grey |
| de Sena-2019[25] | Green | Green | Green | Green | Green | Green | Green | Green | Green | Green | Red | Grey |

Green – yes (low risk of bias); Red – no (higher risk of bias); Orange – unclear; Grey – not applicable; Assessed according to the Joanna Briggs Institute Case Series tool[17] for single arm studies; The appropriateness of analysis was considered appropriate for all studies given that the individual patient data were re-analysed as part of these meta-analyses; PQ – primaquine.

**Figure S3. Scatter plot of primaquine total dose (mg/kg) by A) age (years) and B) weight (kg) categorised by presence of recurrent parasitaemia between the day after the last primaquine dose to 150 days later**

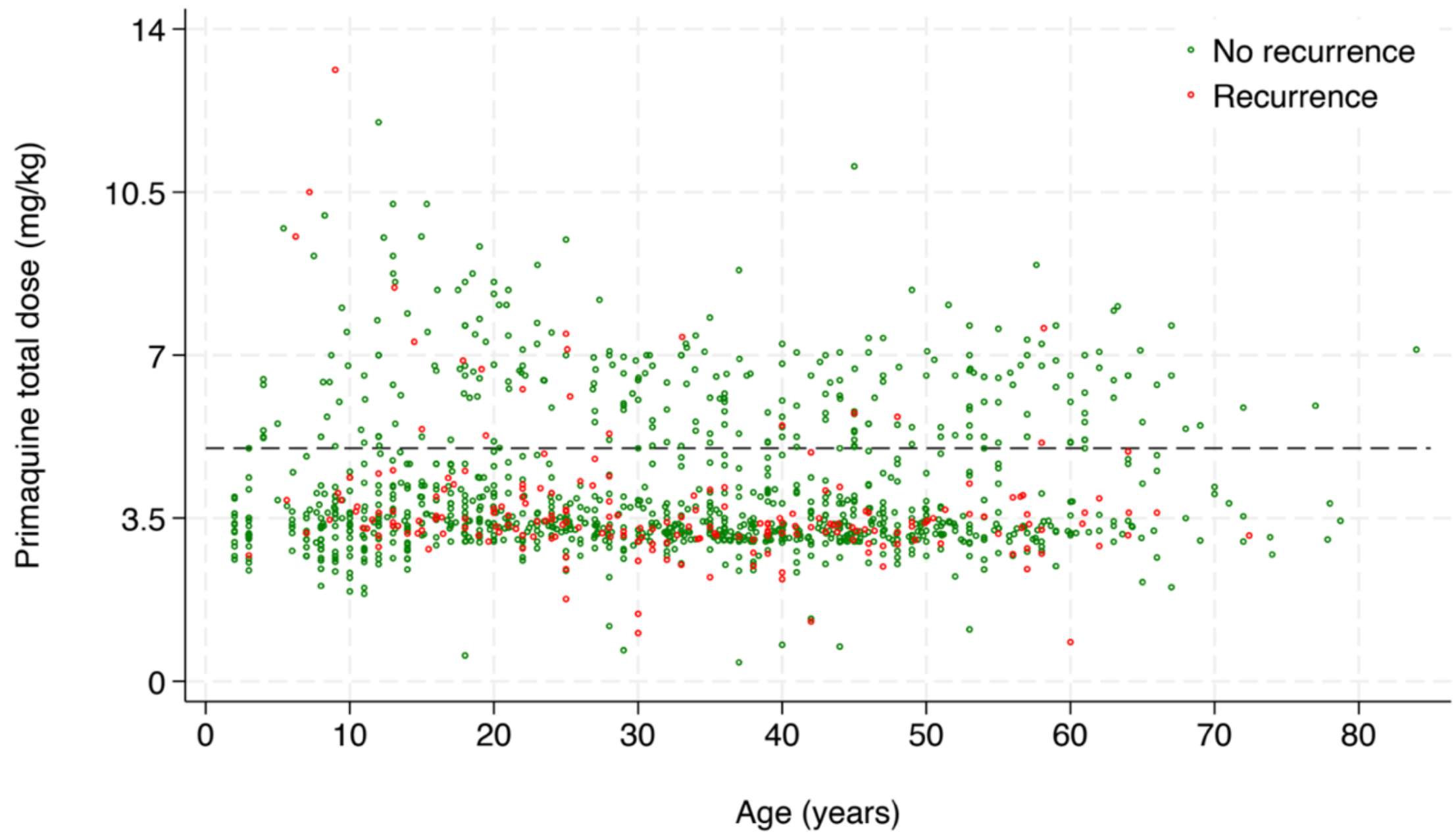


A)

Dotted line at 5 mg/kg, splitting data into low (2-<5 mg/kg) and high total dose primaquine (≥5 mg/kg)

B)

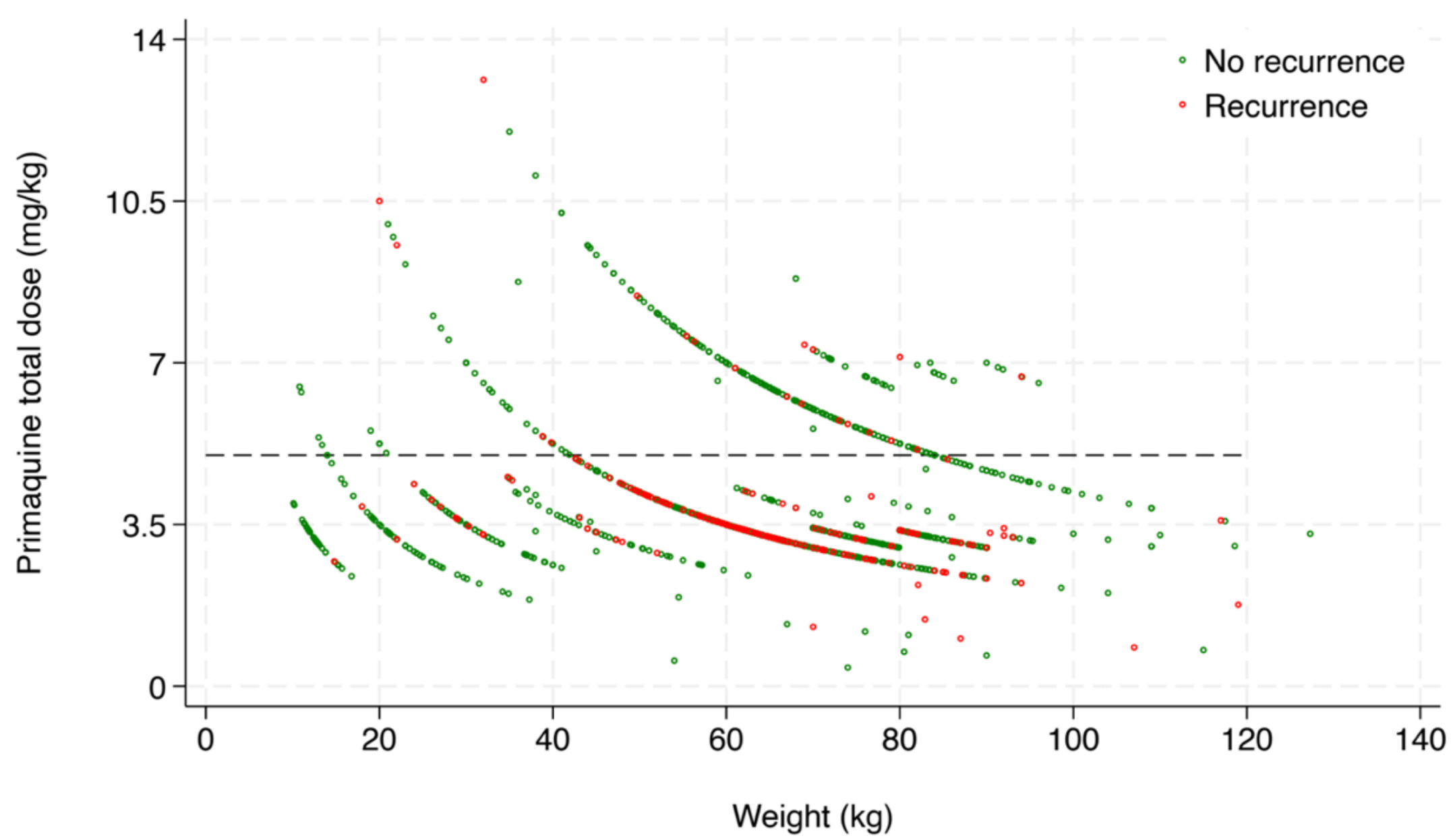


Dotted line at 5 mg/kg, splitting data into low (2-<5 mg/kg) and high total dose primaquine (≥5 mg/kg)

**Table S9. Sensitivity analyses for Cox regression analysis between total primaquine dose mg/kg and first recurrent *P. vivax* parasitaemia**

| Total primaquine dose | First recurrence between day 7 to 180* n= 1129 | Patients administered CQ n=1078 | Patients administered CQ and 14-day PQ regimen n= 646 | Patients commencing PQ prior to day 7 n=1122 | Studies with follow up ≥180 days n=777 | Without shared frailty n= 1420 | Patients with actual PQ dose recorded n= 953 |
|---|---|---|---|---|---|---|---|
| **None** | Reference | Reference | Reference | Reference | Reference | Reference | Reference |
| **Low** | 0.34 (0.23-0.49) | 0.34 (0.23-0.50) | 0.30 (0.21-0.44) | 0.34 (0.23-0.49) | 0.35 (0.24-0.51) | 0.32 (0.24-0.42) | 0.31 (0.08-1.21) |
| **High** | 0.15 (0.05-0.42) | 0.10 (0.05-0.21) | 0.09 (0.04-0.20) | 0.15 (0.05-0.43) | 0.17 (0.06-0.53) | 0.08 (0.05-0.12) | 0.12 (0.03-0.49) |

CI- confidence interval; CQ – chloroquine; PQ- primaquine; Sensitivity analyses for multivariable Cox regression model (AHR [95% CI]) adjusted for age, sex and baseline parasitaemia with shared frailty unless stated otherwise. *excluding Chamma-Siqueira-2022[29].

**Table S10. Sensitivity analysis removing one study site at a time for relationship between total primaquine mg/kg dose and first recurrent *P. vivax* parasitaemia**

| Variable | Range of AHR | Coefficient of Variation (%) |
|---|---|---|
| *Total primaquine dose* | | |
| None | Reference | - |
| Low | 0.30-0.34 | 3.04 |
| High | 0.10-0.15 | 6.97 |

AHR–Adjusted Hazard Ratio; Sensitivity analysis for multivariable Cox regression model adjusted for age, sex and (log) baseline parasite density with shared frailty for study site.

**Figure S4. Cumulative incidence of first *P. vivax* recurrence in patients administered chloroquine by primaquine total dose**

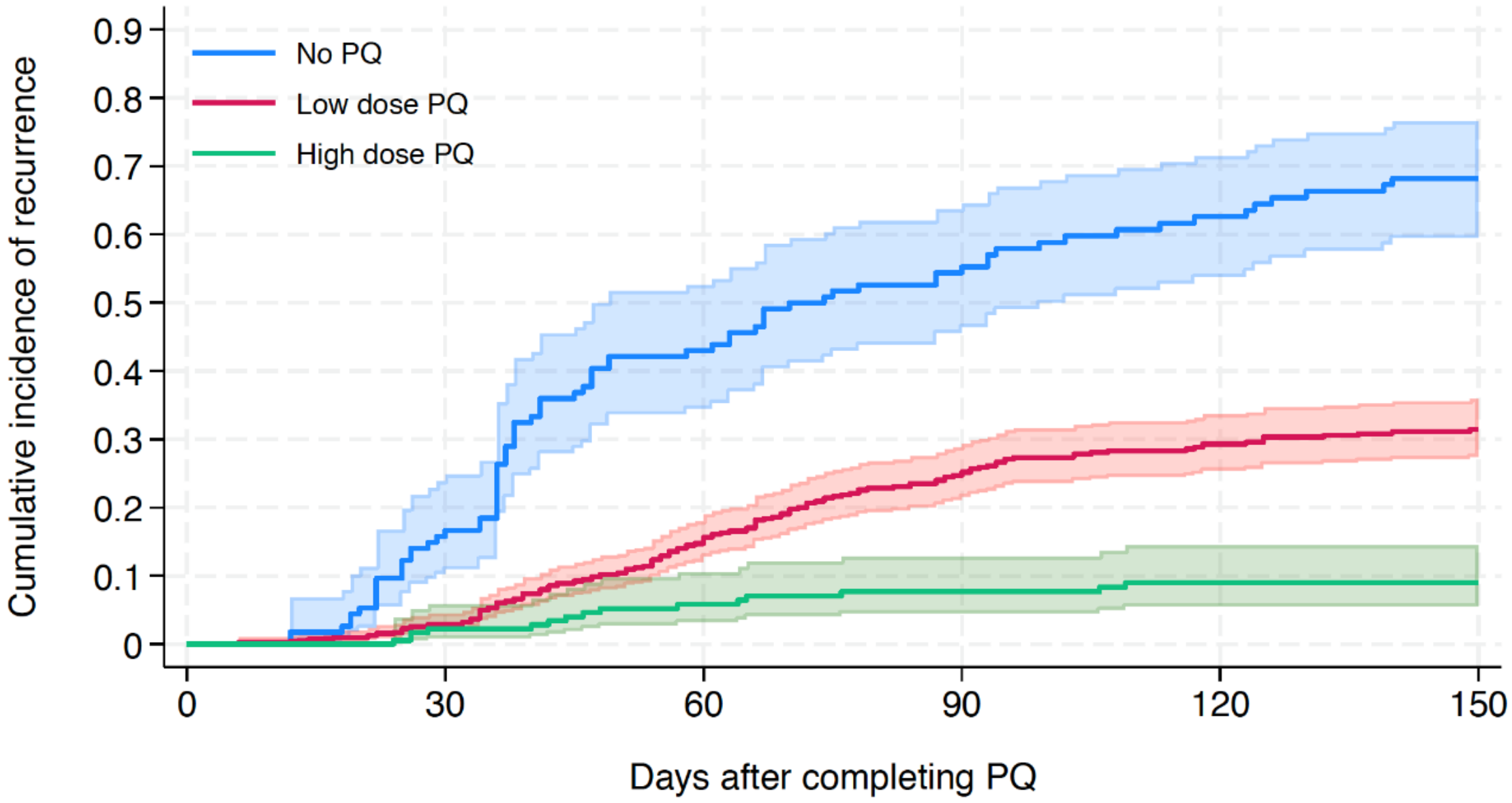


Number at risk

| | | | | | | | | | | | |
|---|---|---|---|---|---|---|---|---|---|---|---|
| No PQ | 117 | (18) | 96 | (31) | 65 | (13) | 52 | (9) | 40 | (6) | 24 |
| Low dose PQ | 771 | (20) | 627 | (63) | 372 | (43) | 316 | (19) | 284 | (8) | 172 |
| High dose PQ | 190 | (4) | 172 | (6) | 154 | (3) | 146 | (2) | 133 | (0) | 62 |

PQ – primaquine; Primaquine categorised as low total mg/kg dose (<5 mg/kg) and high total mg/kg dose (≥5 mg/kg).

**Figure S5. Cumulative incidence of first *P. vivax* recurrence in patients administered chloroquine and a 14-day primaquine regimen by primaquine total dose**

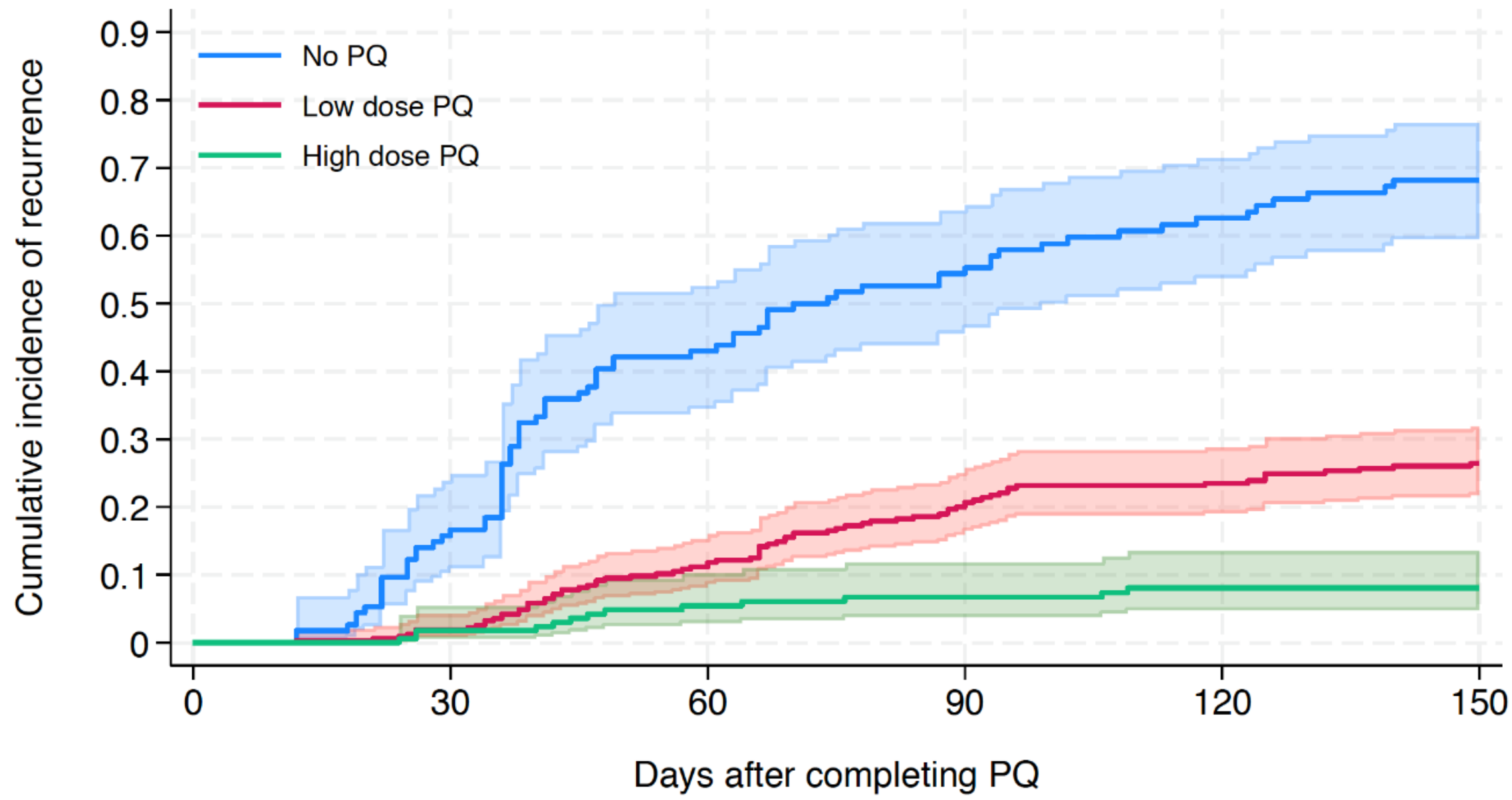


Number at risk

| | | | | | | | | | | | |
|---|---|---|---|---|---|---|---|---|---|---|---|
| No PQ | 117 | (18) | 96 | (31) | 65 | (13) | 52 | (9) | 40 | (6) | 24 |
| Low dose PQ | 350 | (6) | 299 | (28) | 265 | (26) | 230 | (10) | 213 | (8) | 169 |
| High dose PQ | 179 | (3) | 163 | (6) | 150 | (2) | 144 | (2) | 131 | (0) | 62 |

PQ – primaquine; Primaquine categorised as low total mg/kg dose (<5 mg/kg) and high total mg/kg dose (≥5 mg/kg).

**Table S11. Dose groups, number of events and time at risk by study for the efficacy analysis assessing the cumulative incidence of *P. vivax* recurrence within 150 days of the last primaquine dose**

| | Gonzalez-Ceron-2015 | Zuluaga-Idarraga-2016 | Daher-2018 | Ladeia-Andrade-2019 | Llanos-Cuentas-2019 | Lacerda-2019 | Llanos-Cuentas-2014 | de Sena-2019 | Chamma-Siqueira-2022 | Baia-da-Silva-2026 |
|---|---|---|---|---|---|---|---|---|---|---|
| Total included | 88 | 87 | 264 | 94 | 62 | 182 | 55 | 111 | 250 | 212 |
| Primaquine total dose category | | | | | | | | | | |
| No primaquine | 0 (0.0%) | 0 (0.0%) | 0 (0.0%) | 0 (0.0%) | 0 (0.0%) | 92 (50.5%) | 27 (49.1%) | 0 (0.0%) | 0 (0.0%) | 0 (0.0%) |
| Low dose total primaquine (2-<5mg/kg) | 78 (88.6%) | 84 (96.6%) | 264 (100.0%) | 93 (98.9%) | 62 (100.0%) | 90 (49.5%) | 28 (50.9%) | 106 (95.5%) | 152 (60.8%) | 43 (20.3%) |
| High dose total primaquine (≥5mg/kg) | 10 (11.4%) | 3 (3.4%) | 0 (0.0%) | 1 (1.1%) | 0 (0.0%) | 0 (0.0%) | 0 (0.0%) | 5 (4.5%) | 98 (39.2%) | 169 (79.7%) |
| Number of recurrences, n/N (%) | 0 (0.0%) | 18 (20.7%) | 33 (12.5%) | 20 (21.3%) | 18 (29.0%) | 88 (48.4%) | 28 (50.9%) | 2 (1.8%) | 55 (22.0%) | 11 (5.2%) |
| Time at risk (days), median (IQR) | 14 (13-150) | 150 (79-150) | 55 (55-55) | 20 (20-22) | 149 (78-150) | 129 (43-150) | 69 (36-150) | 34 (34-34) | 133 (54-139) | 150 (107-150) |

**Figure S6. Two stage individual patient data meta-analysis comparing the hazard ratio for *P. vivax* recurrence following low total dose primaquine compared with no primaquine in studies randomising patients to these two arms**

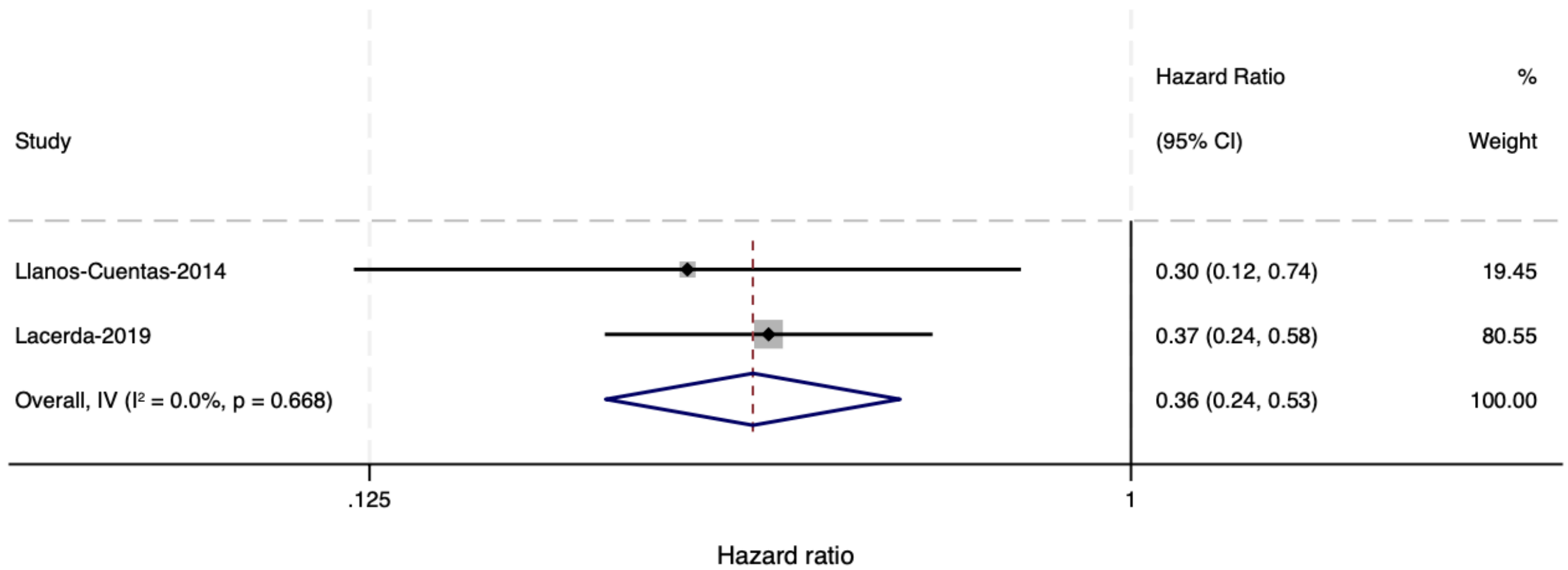


Hazard ratio <1 favours low total dose primaquine. Two stage meta-analysis based on individual patient data for each study that are then aggregated using random effect meta-analysis.

**Figure S7. Cumulative incidence of first *P. vivax* recurrence in patients administered chloroquine and low total dose primaquine by primaquine duration**

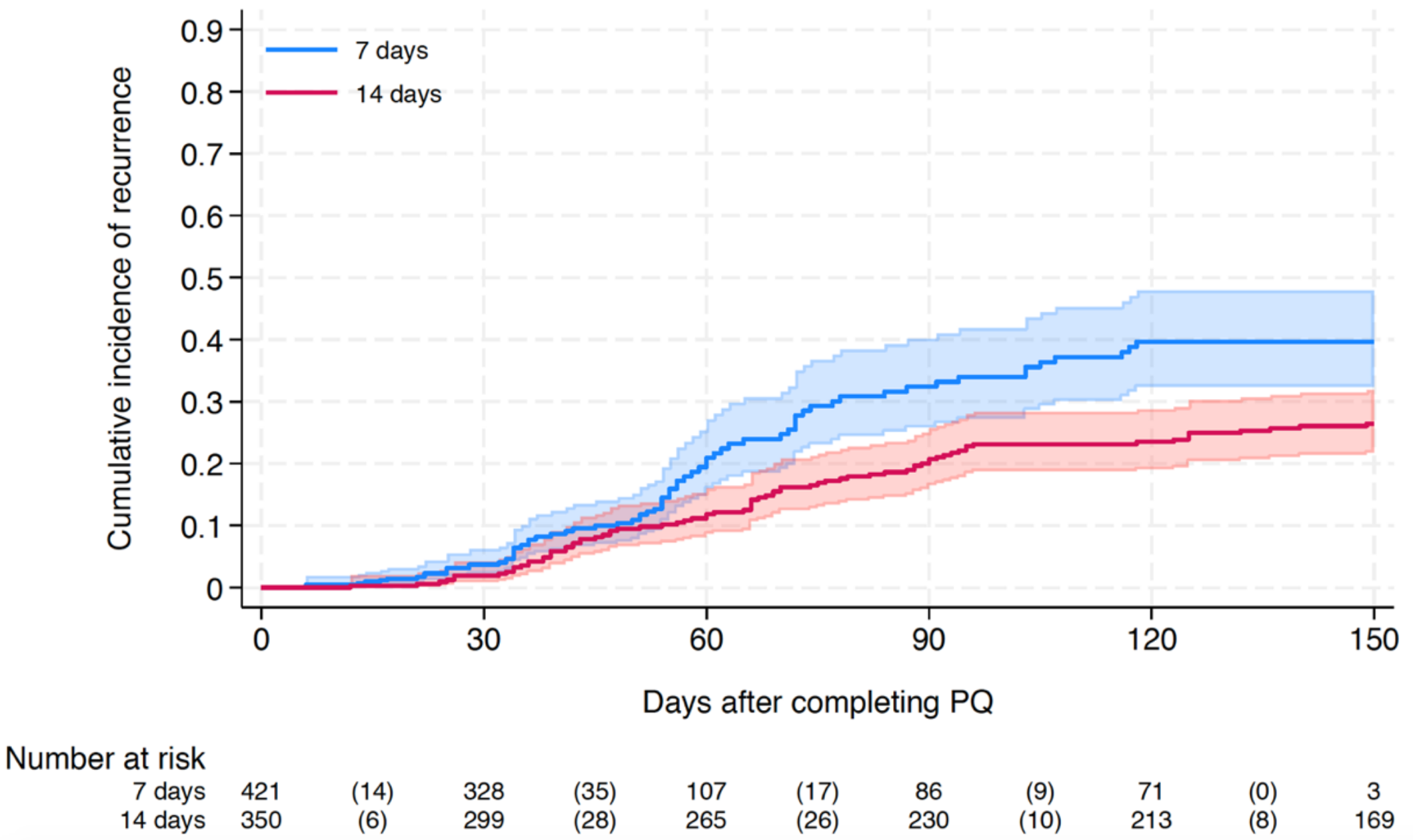


PQ – primaquine; Primaquine categorised as low total mg/kg dose (<5 mg/kg) and high total mg/kg dose (≥5 mg/kg).

**Table S12: Sensitivity analysis of cumulative incidence at day 150 excluding one study at a time in patients administered chloroquine and low total dose primaquine by regimen duration**

| Study excluded | 7-day regimen | 14- day regimen |
|---|---|---|
| Llanos-Cuentas-2013 | 39.7 (32.4,48.0) | 25.7 (20.9,31.4) |
| Gonzalez-Ceron-2015 | 39.7 (32.4,48.0) | 29.1 (24.0,34.9) |
| Zuluaga-Idarraga-2016 | 39.7 (32.4,48.0) | 27.9 (22.4,34.4) |
| Daher-2018 | 41.2 (33.5,49.8) | 26.4 (21.8,31.9) |
| de Sena-2019 | 40.9 (33.6,49.1) | 26.4 (21.8,31.9) |
| Lacerda-2019 | 39.7 (32.4,48.0) | 22.8 (17.7,29.1) |
| Ladeia-Andrade-2019 | 31.9 (24.8,40.3) | 26.4 (21.8,31.9) |
| Llanos-Cuentas-2019 | 39.7 (32.4,48.0) | 25.3 (20.2,31.3) |
| Chamma-Siqueira-2022 | - | 26.5 (21.8,32.0) |
| Siqueira-unpublished2022 | 39.7 (32.4,48.0) | 27.1 (22.2,32.8) |

Cumulative incidence risk % (95% CI)

**Figure S8. Cumulative incidence of first *P. vivax* recurrence in patients administered chloroquine and low total dose primaquine by primaquine duration, excluding Ladeia-Andrade 2019.**

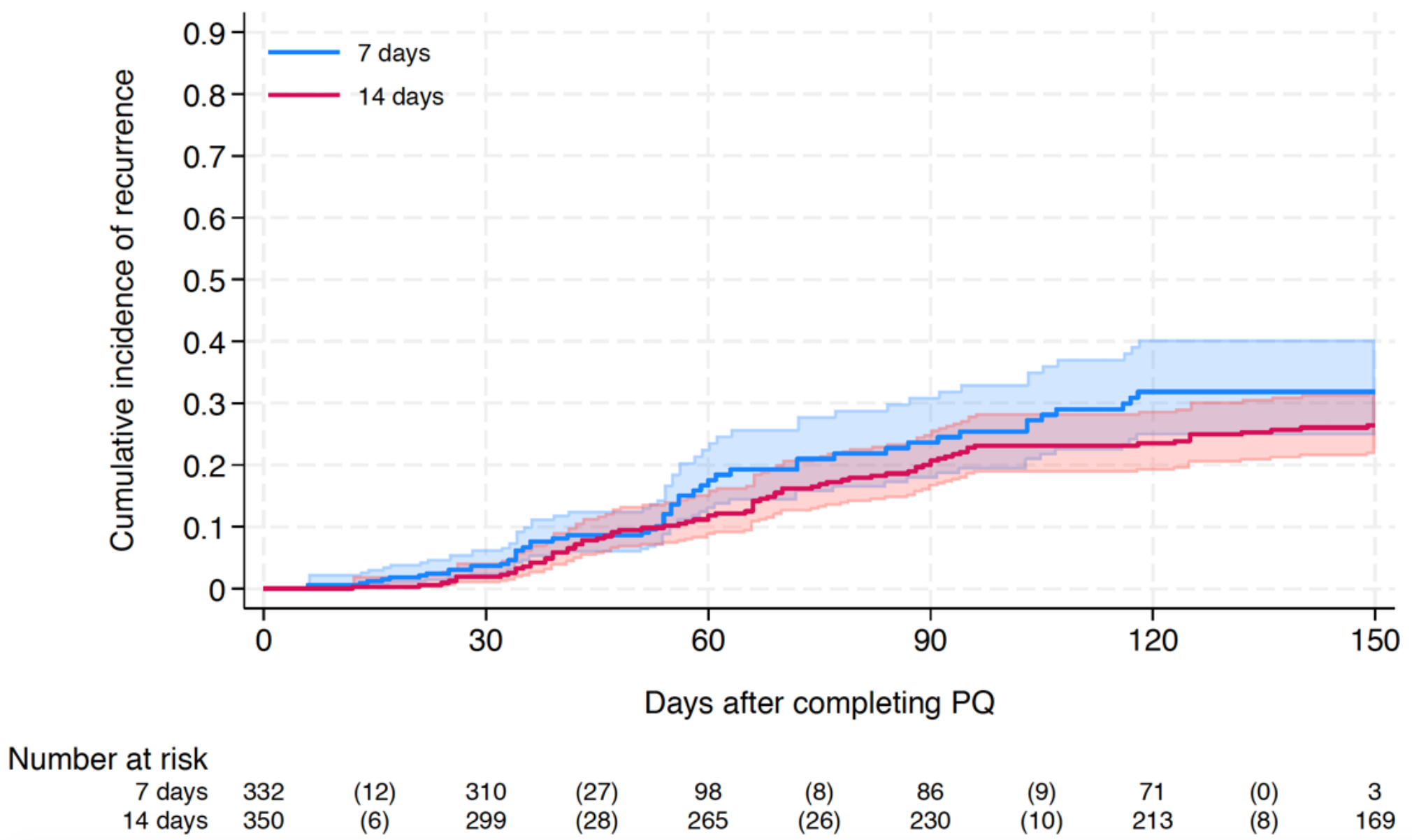

**Table S13. Demographic and baseline characteristics for patients included in the tolerability analysis**

| | Total | Low dose daily primaquine (<0.375 mg/kg/day) | Intermediate dose daily primaquine (≥0.375 & <0.75 mg/kg/day) | High dose daily primaquine (≥0.75 mg/kg/day) |
|---|---|---|---|---|
| | N=489 | N=173 | N=309 | N=7 |
| Age (years) | | | | |
| Median | 35.0 (22.0-47.0) | 36.0 (27.0-46.0) | 36.0 (22.0-48.0) | 11.0 (11.0-15.0) |
| <5 | 1/489 (0.2%) | 1/173 (0.6%) | 0/309 (0.0%) | 0/7 (0.0%) |
| 5-<15 | 46/489 (9.4%) | 10/173 (5.8%) | 31/309 (10.0%) | 5/7 (71.4%) |
| ≥15 | 442/489 (90.4%) | 162/173 (93.6%) | 278/309 (90.0%) | 2/7 (28.6%) |
| Sex | | | | |
| Male | 325/489 (66.5%) | 124/173 (71.7%) | 198/309 (64.1%) | 3/7 (42.9%) |
| Female | 164/489 (33.5%) | 49/173 (28.3%) | 111/309 (35.9%) | 4/7 (57.1%) |
| Enrolment variables | | | | |
| Weight (kg) | 67.0 (58.9-78.0) | 81.5 (63.0-86.5) | 64.9 (56.9-72.0) | 37.0 (35.0-38.8) |
| Presence of recent fever | 481/489 (98.4%) | 171/173 (98.8%) | 303/309 (98.1%) | 7/7 (100.0%) |
| Parasitaemia (parasites per mL) | 3240.0 (1485.0-5514.2) | 3420.0 (1040.0-6461.5) | 3199.0 (1539.9-5120.0) | 5061.6 (2828.1-13301.8) |
| Haemoglobin day 0 (g/dL) | 13.5 (1.7) | 14.4 (1.5) | 13.2 (1.7) | 12.1 (0.6) |
| Schizontocidal treatment | | | | |
| Chloroquine | 378/489 (77.3%) | 140/173 (80.9%) | 232/309 (75.1%) | 6/7 (85.7%) |
| Dihydroartemisinin-piperaquine | 111/489 (22.7%) | 33/173 (19.1%) | 77/309 (24.9%) | 1/7 (14.3%) |
| Daily dose of primaquine given | 0.4 (0.3-0.5) | 0.3 (0.2-0.3) | 0.5 (0.4-0.5) | 0.8 (0.8-0.9) |
| Primaquine duration (days) | | | | |
| 7 | 180/489 (36.8%) | 20/173 (11.6%) | 156/309 (50.5%) | 4/7 (57.1%) |
| 14 | 309/489 (63.2%) | 153/173 (88.4%) | 153/309 (49.5%) | 3/7 (42.9%) |

| | | | | |
|---|---|---|---|---|
| Daily dose primaquine calculation method | | | | |
| Actual dosing | 222/489 (45.4%) | 73/173 (42.2%) | 146/309 (47.2%) | 3/7 (42.9%) |
| Protocol dosing | 267/489 (54.6%) | 100/173 (57.8%) | 163/309 (52.8%) | 4/7 (57.1%) |
| Primaquine start day | | | | |
| Day 0-2 | 489/489 (100.0%) | 173/173 (100.0%) | 309/309 (100.0%) | 7/7 (100.0%) |
| Primaquine supervision | | | | |
| Unsupervised | 222/489 (45.4%) | 73/173 (42.2%) | 146/309 (47.2%) | 3/7 (42.9%) |
| Partially supervised | 86/489 (17.6%) | 18/173 (10.4%) | 68/309 (22.0%) | 0/7 (0.0%) |
| Fully supervised | 181/489 (37.0%) | 82/173 (47.4%) | 95/309 (30.7%) | 4/7 (57.1%) |
| Study duration (days) | | | | |
| 28 | 86/489 (17.6%) | 18/173 (10.4%) | 68/309 (22.0%) | 0/7 (0.0%) |
| 180 | 403/489 (82.4%) | 155/173 (89.6%) | 241/309 (78.0%) | 7/7 (100.0%) |
| Primaquine administered with food | | | | |
| No | 94/267 (35.2%) | 2/100 (2.0%) | 88/163 (54.0%) | 4/4 (100.0%) |
| Recommended | 173/267 (64.8%) | 98/100 (98.0%) | 75/163 (46.0%) | 0/4 (0.0%) |
| Transmission intensity of study site | | | | |
| Moderate | 87/489 (17.8%) | 80/173 (46.2%) | 7/309 (2.3%) | 0/7 (0.0%) |
| High | 402/489 (82.2%) | 93/173 (53.8%) | 302/309 (97.7%) | 7/7 (100.0%) |

**Table S14: Demographic and baseline characteristics for patients with known G6PD activity included in the haematological safety analysis**

| | Total | No primaquine | Low daily dose primaquine (<0.375 mg/kg/day) | Intermediate daily dose primaquine (≥0.375 & <0.75 mg/kg/day) | High daily dose primaquine (≥0.75 mg/kg/day) |
|---|---|---|---|---|---|
| | N=501 | N=119 | N=129 | N=246 | N=7 |
| Age (years) | | | | | |
| Median | 36.0 (23.0-49.0) | 35.0 (26.0-49.0) | 39.0 (29.0-51.0) | 35.0 (20.0-49.0) | 11.0 (11.0-15.0) |
| ≤5 | 32/501 (6.4%) | 0/119 (0.0%) | 0/129 (0.0%) | 27/246 (11.0%) | 5/7 (71.4%) |
| ≥15 | 469/501 (93.6%) | 119/119 (100.0%) | 129/129 (100.0%) | 219/246 (89.0%) | 2/7 (28.6%) |
| Sex | | | | | |
| Male | 340/501 (67.9%) | 82/119 (68.9%) | 100/129 (77.5%) | 155/246 (63.0%) | 3/7 (42.9%) |
| Female | 161/501 (32.1%) | 37/119 (31.1%) | 29/129 (22.5%) | 91/246 (37.0%) | 4/7 (57.1%) |
| Enrolment variables | | | | | |
| Weight (kg) | 66.4 (59.1-77.0) | 64.5 (60.2-72.5) | 82.1 (66.0-88.1) | 64.2 (56.1-71.5) | 37.0 (35.0-38.8) |
| Presence of recent fever | 487/501 (97.2%) | 111/119 (93.3%) | 127/129 (98.4%) | 242/246 (98.4%) | 7/7 (100.0%) |
| Parasitaemia (parasites per mL) | 3737.6 (1562.0-7034.0) | 4750.0 (1633.0-10243.0) | 3597.0 (1261.9-7937.0) | 3329.0 (1514.5-5810.2) | 5061.6 (2828.1-13301.8) |
| Haemoglobin day 0 (g/dL) | 13.4 (1.7) | 12.9 (1.4) | 14.0 (1.7) | 13.3 (1.7) | 12.1 (0.6) |
| Schizontocidal treatment | | | | | |
| Chloroquine | 392/501 (78.2%) | 119/119 (100.0%) | 98/129 (76.0%) | 169/246 (68.7%) | 6/7 (85.7%) |
| Dihydroartemisinin-piperaquine | 109/501 (21.8%) | 0/119 (0.0%) | 31/129 (24.0%) | 77/246 (31.3%) | 1/7 (14.3%) |
| Total dose of primaquine given | 4.7 (3.3-6.0) | | 3.9 (3.2-4.8) | 5.5 (3.4-6.6) | 5.4 (2.8-12.0) |
| Primaquine duration (days) | | | | | |
| 7 | 102/381 (26.8%) | | 4/129 (3.1%) | 94/245 (38.4%) | 4/7 (57.1%) |
| 14 | 279/381 (73.2%) | | 125/129 (96.9%) | 151/245 (61.6%) | 3/7 (42.9%) |
| Total dose primaquine calculation method | | | | | |

| | | | | | |
|---|---|---|---|---|---|
| Actual dosing | 324/382 (84.8%) | | 73/129 (56.6%) | 244/246 (99.2%) | 7/7 (100.0%) |
| Protocol dosing | 58/382 (15.2%) | | 56/129 (43.4%) | 2/246 (0.8%) | 0/7 (0.0%) |
| Primaquine start day | | | | | |
| Day 0-2 | 360/501 (71.9%) | 0/119 (0.0%) | 126/129 (97.7%) | 227/246 (92.3%) | 7/7 (100.0%) |
| Day 3-14 | 22/501 (4.4%) | 0/119 (0.0%) | 3/129 (2.3%) | 19/246 (7.7%) | 0/7 (0.0%) |
| >Day 14 | 119/501 (23.8%) | 119/119 (100.0%) | 0/129 (0.0%) | 0/246 (0.0%) | 0/7 (0.0%) |
| Primaquine supervision | | | | | |
| Unsupervised | 219/501 (43.7%) | 0/119 (0.0%) | 69/129 (53.5%) | 147/246 (59.8%) | 3/7 (42.9%) |
| Partially supervised | 177/501 (35.3%) | 119/119 (100.0%) | 56/129 (43.4%) | 2/246 (0.8%) | 0/7 (0.0%) |
| Fully supervised | 105/501 (21.0%) | 0/119 (0.0%) | 4/129 (3.1%) | 97/246 (39.4%) | 4/7 (57.1%) |
| Study duration (days) | | | | | |
| 168 | 20/501 (4.0%) | 0/119 (0.0%) | 3/129 (2.3%) | 17/246 (6.9%) | 0/7 (0.0%) |
| 180 | 481/501 (96.0%) | 119/119 (100.0%) | 126/129 (97.7%) | 229/246 (93.1%) | 7/7 (100.0%) |
| Primaquine administered with food | | | | | |
| No | 89/266 (33.5%) | 0/119 (0.0%) | 2/58 (3.4%) | 83/85 (97.6%) | 4/4 (100.0%) |
| Yes | 177/266 (66.5%) | 119/119 (100.0%) | 56/58 (96.6%) | 2/85 (2.4%) | 0/4 (0.0%) |
| Transmission intensity of study site | | | | | |
| High | 501/501 (100.0%) | 119/119 (100.0%) | 129/129 (100.0%) | 246/246 (100.0%) | 7/7 (100.0%) |

**Figure S9. Aggregated data meta-analysis of trials comparing the risk of *P. vivax* recurrence following high versus low total dose primaquine undertaken in the Americas**

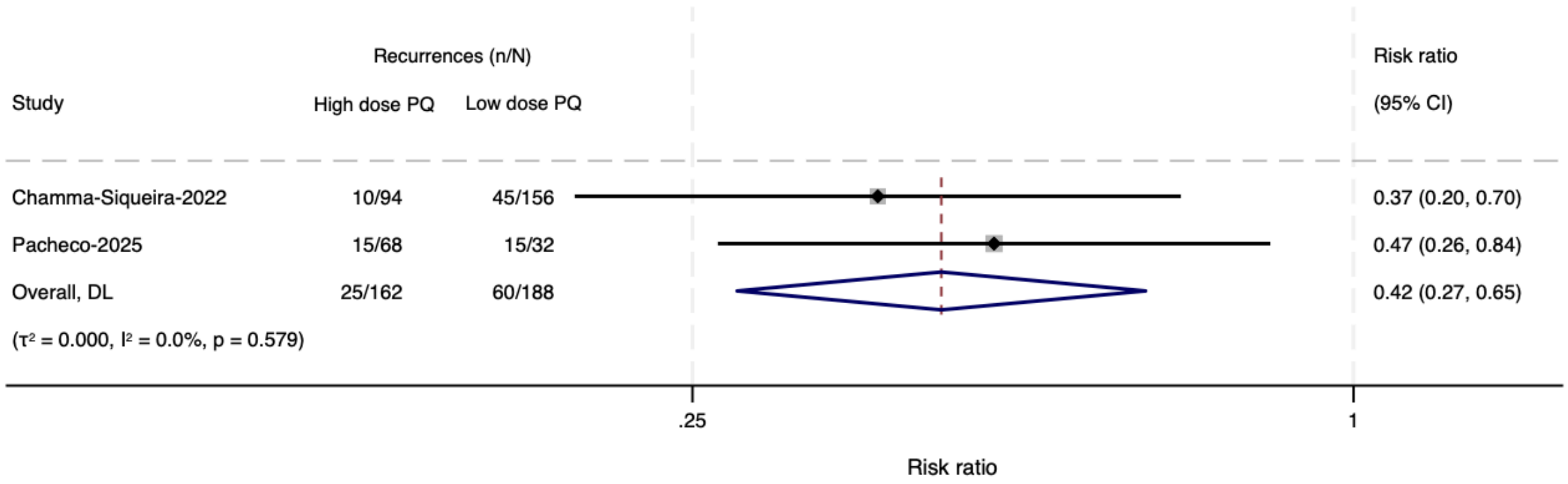


Risk ratio <1 favours high over low total dose primaquine; Aggregated data meta-analysis based on pooled risk ratios that were derived from the number of recurrences in the low or high total dose arms, with the total number of patients in those arms as the denominator.

## References S1


39. Bergonzoli G, Rivers Cuadra JC. [Therapeutic efficacy of different antimalarial regimens in the Costa Rica-Nicaragua border region]. *Rev Panam Salud Publica* 2000; **7**(6): 366-70.
40. Villalobos-Salcedo JM, Tada MS, Kimura E, Menezes MJ, Pereira da Silva LH. In-vivo sensitivity of Plasmodium vivax isolates from Rond nia (western Amazon region, Brazil) to regimens including chloroquine and primaquine. *Ann Trop Med Parasitol* 2000; **94**(8): 749-58.
41. Abdon NP, Pinto AY, das Silva Rdo S, de Souza JM. [Assessment of the response to reduced treatment schemes for vivax malaria]. *Rev Soc Bras Med Trop* 2001; **34**(4): 343-8.
42. Duarte EC, Pang LW, Ribeiro LC, Fontes CJ. Association of subtherapeutic dosages of a standard drug regimen with failures in preventing relapses of vivax malaria. *Am J Trop Med Hyg* 2001; **65**(5): 471-6.
43. Solari Soto L, Soto Tarazona AR, Mendoza Requena D, Llanos Cuentas EA. Ensayo clinico del tratamiento de la malaria vivax con esquema acortado de primaquina comparado con el esquema tradicional. *Rev Soc Peru Med Interna* 2002; **15**(4): 197-9.
44. da Silva Rdo S, Pinto AY, Calvosa VS, de Souza JM. [Short course schemes for vivax malaria treatment]. *Rev Soc Bras Med Trop* 2003; **36**(2): 235-9.
45. Machado RL, de Figuereido Filho AF, Calvosa VS, Figueredo MC, Nascimento JM, Povoa MM. Correlation between Plasmodium vivax variants in Belem, Para State, Brazil and symptoms and clearance of parasitaemia. *Braz J Infect Dis* 2003; **7**(3): 175-7.
46. Pinto AY, Azevedo CH, da Silva JB, de Souza JM. Assessment of chloroquine single dose treatment of malaria due to Plasmodium vivax in Brazilian Amazon. *Rev Inst Med Trop Sao Paulo* 2003; **45**(6): 327-31.
47. Alvarez G, Pineros JG, Tobon A, et al. Efficacy of three chloroquine-primaquine regimens for treatment of Plasmodium vivax malaria in Colombia. *Am J Trop Med Hyg* 2006; **75**(4): 605-9.
48. Carmona-Fonseca J, Alvarez G. [Plasmodium vivax malaria: treatment of primary attacks with primaquine, in three different doses, and a fixed dose of chloroquine, Antioquia, Colombia, 2003-2004]. *Biomedica* 2006; **26**(3): 353-65.
49. Perez MA, Cortes LJ, Guerra AP, Knudson A, Usta C, Nicholls RS. [Efficacy of the amodiaquine+sulfadoxine-pyrimethamine combination and of chloroquine for the treatment of malaria in Cordoba, Colombia, 2006]. *Biomedica* 2008; **28**(1): 148-59.
50. Carmona-Fonseca J, Maestre A. Prevention of Plasmodium vivax malaria recurrence: efficacy of the standard total dose of primaquine administered over 3 days. *Acta Trop* 2009; **112**(2): 188-92.
51. Orjuela-Sanchez P, da Silva NS, da Silva-Nunes M, Ferreira MU. Recurrent parasitemias and population dynamics of Plasmodium vivax polymorphisms in rural Amazonia. *Am J Trop Med Hyg* 2009; **81**(6): 961-8.
52. Carmona-Fonseca J. Vivax malaria in children: Recurrences with standard total dose of primaquine administered in 3 vs. 7 days *Iatreia* 2010; **23**(1): 10-20.
53. Van den Eede P, Soto-Calle VE, Delgado C, et al. Plasmodium vivax sub-patent infections after radical treatment are common in Peruvian patients: results of a 1-year prospective cohort study. *PLoS One* 2011; **6**(1): e16257.
54. Eibach D, Ceron N, Krishnalall K, et al. Therapeutic efficacy of artemether-lumefantrine for Plasmodium vivax infections in a prospective study in Guyana. *Malar J* 2012; **11**: 347.
55. Graf PC, Durand S, Alvarez Antonio C, et al. Failure of Supervised Chloroquine and Primaquine Regimen for the Treatment of Plasmodium vivax in the Peruvian Amazon. *Malar Res Treat* 2012; **2012**: 936067.
56. Pedro RS, Guaraldo L, Campos DP, Costa AP, Daniel-Ribeiro CT, Brasil P. Plasmodium vivax malaria relapses at a travel medicine centre in Rio de Janeiro, a non-endemic area in Brazil. *Malar J* 2012; **11**: 245.

57. Delgado-Ratto C, Soto-Calle VE, Van den Eede P, et al. Population structure and spatio-temporal transmission dynamics of Plasmodium vivax after radical cure treatment in a rural village of the Peruvian Amazon. *Malar J* 2014; **13**: 8.
58. Negreiros S, Farias S, Viana GM, et al. Efficacy of Chloroquine and Primaquine for the Treatment of Uncomplicated Plasmodium vivax Malaria in Cruzeiro do Sul, Brazil. *Am J Trop Med Hyg* 2016; **95**(5): 1061-8.
59. Mac Donald-Ottevanger MS, Adhin MR, Jitan JK, Bretas G, Vreden SG. Primaquine double dose for 7 days is inferior to single-dose treatment for 14 days in preventing Plasmodium vivax recurrent episodes in Suriname. *Infect Drug Resist* 2018; **11**: 3-8.
60. Brasil LW, Rodrigues-Soares F, Santoro AB, et al. CYP2D6 activity and the risk of recurrence of Plasmodium vivax malaria in the Brazilian Amazon: a prospective cohort study. *Malar J* 2018; **17**(1): 57.
61. Mesa-Echeverry E, Niebles-Bolivar M, Tobon-Castano A. Chloroquine-Primaquine Therapeutic Efficacy, Safety, and Plasma Levels in Patients with Uncomplicated Plasmodium vivax Malaria in a Colombian Pacific Region. *Am J Trop Med Hyg* 2019; **100**(1): 72-7.
62. Barbosa L, Brito-Sousa J, Nascimento C, et al. Safety and Efficacy of 3 Alternative Regimens Against Relapsing Plasmodium vivax Malaria in Glucose 6-Phosphate Dehydrogenase-Deficient Patients in the Brazilian Amazon (ALTPRIM). *Clin Infect Dis* 2025; **81**(5): e294-e301.
63. Calderon-Ruiz P, Velez-Tobon G, Bolivar-Hernandez S, Murcia-Montano LM, Tobon-Castano A. Chloroquine-primaquine therapeutic response and safety in patients with uncomplicated Plasmodium vivax malaria in the Colombian Amazon region. *Malar J* 2024; **23**(1): 348.
64. Godinho VKC, Mwangi VI, Morais MC, et al. Differential impact of Tafenoquine and Primaquine on Plasmodium vivax recurrence: the role of CYP2D6 variants. *Antimicrob Agents Chemother* 2026; **70**(6): e0179725.
